# Characterization of long COVID temporal sub-phenotypes by distributed representation learning from electronic health record data: a cohort study

**DOI:** 10.1016/j.eclinm.2023.102210

**Published:** 2023-09-14

**Authors:** Arianna Dagliati, Zachary H. Strasser, Zahra Shakeri Hossein Abad, Jeffrey G. Klann, Kavishwar B. Wagholikar, Rebecca Mesa, Shyam Visweswaran, Michele Morris, Yuan Luo, Darren W. Henderson, Malarkodi Jebathilagam Samayamuthu, Bryce W.Q. Tan, Guillame Verdy, Gilbert S. Omenn, Zongqi Xia, Riccardo Bellazzi, The Consortium for Clinical Characterization of COVID-19 by EHR (4CE), James R. Aaron, Giuseppe Agapito, Adem Albayrak, Giuseppe Albi, Mario Alessiani, Anna Alloni, Danilo F. Amendola, François Angoulvant, Li L.L.J. Anthony, Bruce J. Aronow, Fatima Ashraf, Andrew Atz, Paul Avillach, Paula S. Azevedo, James Balshi, Brett K. Beaulieu-Jones, Douglas S. Bell, Antonio Bellasi, Riccardo Bellazzi, Vincent Benoit, Michele Beraghi, José Luis Bernal-Sobrino, Mélodie Bernaux, Romain Bey, Surbhi Bhatnagar, Alvar Blanco-Martínez, Clara-Lea Bonzel, John Booth, Silvano Bosari, Florence T. Bourgeois, Robert L. Bradford, Gabriel A. Brat, Stéphane Bréant, Nicholas W. Brown, Raffaele Bruno, William A. Bryant, Mauro Bucalo, Emily Bucholz, Anita Burgun, Tianxi Cai, Mario Cannataro, Aldo Carmona, Charlotte Caucheteux, Julien Champ, Jin Chen, Krista Y. Chen, Luca Chiovato, Lorenzo Chiudinelli, Kelly Cho, James J. Cimino, Tiago K. Colicchio, Sylvie Cormont, Sébastien Cossin, Jean B. Craig, Juan Luis Cruz-Bermúdez, Jaime Cruz-Rojo, Arianna Dagliati, Mohamad Daniar, Christel Daniel, Priyam Das, Batsal Devkota, Audrey Dionne, Rui Duan, Julien Dubiel, Scott L. DuVall, Loic Esteve, Hossein Estiri, Shirley Fan, Robert W. Follett, Thomas Ganslandt, Noelia García- Barrio, Lana X. Garmire, Nils Gehlenborg, Emily J. Getzen, Alon Geva, Tobias Gradinger, Alexandre Gramfort, Romain Griffier, Nicolas Griffon, Olivier Grisel, Alba Gutiérrez-Sacristán, Larry Han, David A. Hanauer, Christian Haverkamp, Derek Y. Hazard, Bing He, Darren W. Henderson, Martin Hilka, Yuk-Lam Ho, John H. Holmes, Chuan Hong, Kenneth M. Huling, Meghan R. Hutch, Richard W. Issitt, Anne Sophie Jannot, Vianney Jouhet, Ramakanth Kavuluru, Mark S. Keller, Chris J. Kennedy, Daniel A. Key, Katie Kirchoff, Jeffrey G. Klann, Isaac S. Kohane, Ian D. Krantz, Detlef Kraska, Ashok K. Krishnamurthy, Sehi L'Yi, Trang T. Le, Judith Leblanc, Guillaume Lemaitre, Leslie Lenert, Damien Leprovost, Molei Liu, Ne Hooi Will Loh, Qi Long, Sara Lozano-Zahonero, Yuan Luo, Kristine E. Lynch, Sadiqa Mahmood, Sarah E. Maidlow, Adeline Makoudjou, Alberto Malovini, Kenneth D. Mandl, Chengsheng Mao, Anupama Maram, Patricia Martel, Marcelo R. Martins, Jayson S. Marwaha, Aaron J. Masino, Maria Mazzitelli, Arthur Mensch, Marianna Milano, Marcos F. Minicucci, Bertrand Moal, Taha Mohseni Ahooyi, Jason H. Moore, Cinta Moraleda, Jeffrey S. Morris, Michele Morris, Karyn L. Moshal, Sajad Mousavi, Danielle L. Mowery, Douglas A. Murad, Shawn N. Murphy, Thomas P. Naughton, Carlos Tadeu Breda Neto, Antoine Neuraz, Jane Newburger, Kee Yuan Ngiam, Wanjiku F.M. Njoroge, James B. Norman, Jihad Obeid, Marina P. Okoshi, Karen L. Olson, Gilbert S. Omenn, Nina Orlova, Brian D. Ostasiewski, Nathan P. Palmer, Nicolas Paris, Lav P. Patel, Miguel Pedrera-Jiménez, Emily R. Pfaff, Ashley C. Pfaff, Danielle Pillion, Sara Pizzimenti, Hans U. Prokosch, Robson A. Prudente, Andrea Prunotto, Víctor Quirós-González, Rachel B. Ramoni, Maryna Raskin, Siegbert Rieg, Gustavo Roig-Domínguez, Pablo Rojo, Paula Rubio-Mayo, Paolo Sacchi, Carlos Sáez, Elisa Salamanca, Malarkodi Jebathilagam Samayamuthu, L. Nelson Sanchez-Pinto, Arnaud Sandrin, Nandhini Santhanam, Janaina C.C. Santos, Fernando J. Sanz Vidorreta, Maria Savino, Emily R. Schriver, Petra Schubert, Juergen Schuettler, Luigia Scudeller, Neil J. Sebire, Pablo Serrano-Balazote, Patricia Serre, Arnaud Serret-Larmande, Mohsin Shah, Zahra Shakeri Hossein Abad, Domenick Silvio, Piotr Sliz, Jiyeon Son, Charles Sonday, Andrew M. South, Anastasia Spiridou, Zachary H. Strasser, Amelia L.M. Tan, Bryce W.Q. Tan, Byorn W.L. Tan, Suzana E. Tanni, Deanne M. Taylor, Ana I. Terriza-Torres, Valentina Tibollo, Patric Tippmann, Emma M.S. Toh, Carlo Torti, Enrico M. Trecarichi, Yi-Ju Tseng, Andrew K. Vallejos, Gael Varoquaux, Margaret E. Vella, Guillaume Verdy, Jill-Jênn Vie, Shyam Visweswaran, Michele Vitacca, Kavishwar B. Wagholikar, Lemuel R. Waitman, Xuan Wang, Demian Wassermann, Griffin M. Weber, Martin Wolkewitz, Scott Wong, Zongqi Xia, Xin Xiong, Ye Ye, Nadir Yehya, William Yuan, Alberto Zambelli, Harrison G. Zhang, Daniela Zo¨ller, Valentina Zuccaro, Chiara Zucco, Shawn N. Murphy, John H. Holmes, Hossein Estiri

**Affiliations:** a Department of Electrical Computer and Biomedical Engineering, University of Pavia, Pavia, Italy; b Department of Medicine, Massachusetts General Hospital, Boston, United States; c University of Toronto, Dalla Lana School of Public Health, Toronto, Canada; d Department of Biomedical Informatics, University of Pittsburgh, Pittsburgh, United States; e Department of Preventive Medicine, Northwestern University, Chicago, United States; f University of Kentucky, Center for Clinical and Translational Science, Lexington, United States; g National University Hospital, Singapore Department of Medicine, Singapore; h Bordeaux University Hospital, IAM Unit, Bordeaux, France; i University of Michigan, Department of Computational Medicine and Bioinformatics, Internal Medicine, Human Genetics, and School of Public Health, Ann Arbor, United States; j University of Pittsburgh Department of Neurology, Pittsburgh, United States; k University of Pennsylvania Perelman School of Medicine, Department of Biostatistics, Epidemiology, and Informatics, Institute for Biomedical Informatics, Philadelphia, United States; l Department of Biomedical Informatics, Harvard Medical School, Boston, United States; m Department of Neurology, Massachusetts General Hospital, Boston, United States

**Keywords:** Post-acute sequelae of SARS-CoV-2, PASC, COVID-19, SARS-CoV-2, Electronic health records

## Abstract

**Background:**

Characterizing Post-Acute Sequelae of COVID (SARS-CoV-2 Infection), or *PASC* has been challenging due to the multitude of sub-phenotypes, temporal attributes, and definitions. Scalable characterization of PASC sub-phenotypes can enhance screening capacities, disease management, and treatment planning.

**Methods:**

We conducted a retrospective multi-centre observational cohort study, leveraging longitudinal electronic health record (EHR) data of 30,422 patients from three healthcare systems in the Consortium for the Clinical Characterization of COVID-19 by EHR (4CE). From the total cohort, we applied a deductive approach on 12,424 individuals with follow-up data and developed a distributed representation learning process for providing augmented definitions for PASC sub-phenotypes.

**Findings:**

Our framework characterized seven PASC sub-phenotypes. We estimated that on average 15.7% of the hospitalized COVID-19 patients were likely to suffer from at least one PASC symptom and almost 5.98%, on average, had multiple symptoms. Joint pain and dyspnea had the highest prevalence, with an average prevalence of 5.45% and 4.53%, respectively.

**Interpretation:**

We provided a scalable framework to every participating healthcare system for estimating PASC sub-phenotypes prevalence and temporal attributes, thus developing a unified model that characterizes augmented sub-phenotypes across the different systems.

**Funding:**

Authors are supported by 10.13039/100000060National Institute of Allergy and Infectious Diseases, 10.13039/100000049National Institute on Aging, 10.13039/100006108National Center for Advancing Translational Sciences, 10.13039/501100001349National Medical Research Council, 10.13039/100000065National Institute of Neurological Disorders and Stroke, 10.13039/501100000780European Union, 10.13039/100000002National Institutes of Health, 10.13039/100006108National Center for Advancing Translational Sciences.

**Research in Context**

**Evidence before this study**

The World Health Organization offers a widely accepted definition of PASC, stating that it involves ongoing or recurring symptoms that persist for three months after the initial acute infection, and cannot be attributed to an alternative diagnosis. Over 10,500 articles in the NCBI hub, which provides current research updates on COVID-19, have covered topics related to Post-Acute Sequelae of SARS-CoV-2 (PASC), discuss various clusters of symptoms observed in patients infected with the virus, such as anosmia, myocarditis, and fatigue. Within this collection of literature, both prospective and retrospective cohort studies have tracked the progression of persistent PASC symptoms and estimated their prevalence in individuals.

**Added value of this study**

The presented framework enhances PASC definition via a deductive framework that integrate current literature, clinical knowledge and machine learning algorithms. A distributed learning approach based on EHR longitudinal data provides a robust temporal characterization of PASC sub-phenotypes and their symptoms with a fine temporal granularity.

**Implications of all the available evidence**

The systematic characterization of PASC sub-phenotypes will assist researchers in identifying specific groups for further investigation. By estimating the temporal distribution of PASC symptoms, interventions can be implemented promptly and effectively, enabling clinicians to identify evolving sub-phenotypes of PASC and provide personalized patient care. This will also aid healthcare providers in planning treatment and management plans tailored to the unique symptoms and traits of each sub-phenotype. Implementing a scalable approach to characterize PASC sub-phenotypes can empower public health officials to identify risk factors and develop strategies aimed at preventing or reducing the impact of PASC in the broader population.

## Introduction

The lingering long-term effect of SARS-CoV-2 infection, known as the post-acute sequelae of SARS-CoV-2 (PASC), commonly referred to as “long COVID”, is an important area of investigation in the scientific community. Early in the pandemic it was discovered that a large proportion of COVID-19 patients had wide-ranging, persistent symptoms after the acute phase,^1^ including effects on cardiovascular, musculoskeletal, psychiatric, respiratory, and neurological systems.^2^, ^3^, ^4^ The impact of this growing cohort of patients on the healthcare system and broader society has yet to be fully realized. Despite its highly variable impact on patients, PASC healthcare processes are poorly documented. Since October 2021, the Centers for Disease Control and Prevention has proposed the International Classification of Diseases (ICD-10-CM) diagnosis code U09.9 to document unspecified post-COVID-19 conditions. The use of the U09.9 code encompasses an array of conditions pertaining to post-acute conditions.^5^

Robust validated frameworks for defining PASC sub-phenotypes (see Appendix Glossary) are lacking, resulting in inconsistent definitions of patient cohorts in studies and ambiguities for health authorities in patient screening and resource allocation. Scalable frameworks for screening patients who might be suffering from PASC problems, with higher precision diagnostics than are currently available, would enable public health authorities to improve resource allocations and preventive interventions, and researchers to identify risk factors for developing specific PASC sub-phenotypes.

Electronic health records (EHRs) hold valuable structured and unstructured patient data. Despite potential noise and errors, structured EHR data offer low-cost and feasible solutions for developing screening tools for PASC.^6^ By appropriately processing and analyzing structured EHR data, valuable insights can be obtained. These methodologies can be easily implemented and validated across diverse patient populations and healthcare settings due to the widespread availability of structured EHR data.^7^

The Consortium for the Clinical Characterization of COVID-19 by EHR (4CE)^8^ has established an international platform that enables EHR data-driven studies to inform clinicians, epidemiologists, and the general public about COVID-19 using data acquired through the healthcare process. This study leverages the 4CE framework, which provides its members with common data models, extract-transform-load (ETL) procedures, and data quality checks to extract standardized EHR datasets for secondary analysis.

Since the first clinical observations, several efforts have been made to define PASC, understand its epidemiology, and provide unambiguous phenotyping with use of comprehensive data. However, the exact definitions of PASC are still evolving. One widely used definition from the World Health Organization defines PASC as ongoing or recurrent symptoms three months after the initial acute infection that cannot be explained by an alternative diagnosis and that has an impact on the patient's life.^9^ The definition includes a wide range of symptoms; however, this definition may become further refined.

Since April 2020, the literature hub for tracking up-to-date published research on COVID-19 has reported PASC-related topics coverage by over 10,500 articles [https://www.ncbi.nlm.nih.gov/research/coronavirus/]. More than 40 percent of these articles describe symptom clusters observed in patients infected with SARS-CoV-2 after fixed periods, including anosmia, myocarditis, and fatigue among those with the highest occurrence in the literature (Figure S1). While the key aspects of PASC and its prevalence have been defined, some important aspects remain undefined, such as actual boundaries of symptom constellations, and its similarity to other viral and non-viral diseases.^10^

Prospective and retrospective cohort studies tracked the progression of persisting PASC symptoms and estimated prevalence among individuals that likely have another respiratory infection.^11^ International studies identified robust conditions associated with PASC, emphasizing cardiovascular and neurological phenotype profiles of PASC.^12^ Studies based on a prospective cohort of patients gave insight about the evolution of symptoms of post COVID-19 and offer insight into the etiologies and mechanisms underlying this disease. This study^13^ depicts the slow recovery from the acute infection and indicates that the prevalence for most symptoms decreased over time before plateauing between six and eight months after onset.

National studies^14^ exploited primary care cohorts and used ontological methods to create PASC phenotypes that can be shared and inform further research.

Despite progress in understanding the PASC disease spectrum, the majority of current studies still lack evidence from EHR data to validate clinical observations and assumptions and support unambiguous PASC phenotyping, and to identify how PASC clusters of symptoms evolve over time. Thygesen et al.^15^ characterized COVID-19 trajectories, and defined and validated 10 phenotypes on the basis of the integration of eight linked National Health Service datasets for people in England. Comparable attempts to define and validate PASC phenotypes through EHR data, supported by informatics analytic frameworks, are still very limited. In^16^ a method for computationally modeling PASC phenotype data based on EHRs was presented. A US-based cohort of more than 2000 COVID-19 patients was analyzed with unsupervised clustering approaches, from which authors defined six clusters of symptoms suggesting PASC. These features, however, were not longitudinally analyzed as “evolving symptoms in time” nor was a deductive schema (see Appendix Glossary) for defining clinically relevant PASC sub-phenotypes provided. In^17^ authors exploited EHR data across the UK and Hong Kong to assess consistently higher risk of diseases involving multiple-organ systems, cardiovascular, and all-cause mortality amongst patients with COVID-19 and provided robust evidence on PASC risk and potential delayed sequelae of which clinicians should be informed.

Identifying sub-phenotypes of PASC can help healthcare providers tailor treatment and management plans based on the specific symptoms and characteristics of patients’ sub-phenotype. It can also help improve our understanding of the PASC underlying mechanisms and the development of targeted and effective treatments. Scalable characterization of PASC sub-phenotypes can enable public health officials to identify risk factors and develop strategies to prevent or mitigate the impact of PASC in the general population. The estimate of the onset time of specific PASC symptoms and the temporal distribution of each phenotype can reveal deep characteristics of the PASC sub-phenotypes, supporting adequate, timely, and efficient interventions. Our work contributes to developing robust time-based criteria to increase the specificity of the diagnosis of PASC and it provides an evaluation of the temporal distribution of data elements for each PASC sub-phenotype.

## Methods

In this retrospective, multi-database observational cohort study, inpatient electronic medical records were retrieved from three hospital systems from the 4CE sites that participated in this study. We developed a distributed learning process (see Appendix Glossary) that applies a validated ML pipeline, MLHO,^18^ for modeling health outcomes with clinical data from structured EHR data from multiple 4CE sites, to provide augmented definitions for seven PASC sub-phenotypes. The implemented deductive pipeline constructs PASC sub-phenotype definitions, based on clinical knowledge, thanks to a distributed ML framework applied to longitudinal EHR data of 30,422 multinational patients within the 4CE.

### Data source

We conducted the study using the 4CE informatics framework for distributed analysis, facilitated by a common data extract with quality checks, and the computing environment to support the execution of analytic code at individual participating sites [https://github.com/covidclinical]. The 4CE sites extract structured EHR data from patients hospitalized for COVID-19 confirmed by a polymerase chain reaction (PCR) test. A detailed description of the 4CE federated EHR-based study is provided in Weber et al. (see Fig. 4^19^). Each participating institution stored its data on institutional servers, where local analysts run R packages to perform analytics. The patient-level data included demographics, clinical course, diagnosis, medications, and laboratory orders, they were summarized as counts and percentages as shown in Table 1.

**Table 1:** Characteristics of patients from the three healthcare systems.^a^

Number of patients	Total	Hospital system 1	Hospital system 2	Hospital system 3
	(n = 30,422)	(n = 1952)	(n = 8344)	(n = 20,126)
Mean Age, years (SD)	62.3 (21.04)	51.7 (22.0)	61.2 (19.6)	63.8 (21.2)
Median Age, years (IQR)	–	56 (36–68)	63 (48–76)	68 (54–79)
**Era of Diagnosis Count (%)**				
2020Q1	372 (1.2)	–	305 (3.65)	67 (0.33)
2020Q2	2777 (9.1)	44 (2.3)	2330 (27.9)	403 (2.0)
2020Q3	1229 (4.0)	80 (4.1)	329 (3.94)	820 (4.1)
2020Q4	6605 (21.7)	206 (10.6)	1625 (19.5)	4774 (23.7)
2021Q1	4481 (14.7)	200 (10.2)	1576 (18.9)	2705 (13.4)
2021Q2	2147 (7.1)	118 (6.0)	559 (6.70)	1470 (7.3)
2021Q3	3260 (10.7)	745 (38.1)	551 (6.60)	1964 (9.8)
2021Q4	6251 (20.5)	559 (28.6)	1011 (12.1)	4681 (23.3)
2022Q1	3300 (10.8)	–	58 (0.70)	3242 (161.)
**Sex Count (%)**				
Men	15,296 (50.3)	899 (46.1)	4279 (51.3)	10,118 (50.3)
Women	15,124 (49.7)	1051 (53.8)	4065 (48.7)	10,008 (49.7)
**Comorbidities Count (%)**				
Hypertension	13,504 (44.4)	419 (21.5)	3215 (38.5)	9870 (49.0)
Diabetes	7410 (24.4)	260 (13.3)	1738 (20.8)	5412 (26.9)
Cardiovascular diseases	8679 (28.5)	288 (14.8)	2549 (30.5)	5842 (29.0)
Neurological diseases	3093 (10.2)	121 (6.2)	738 (8.8)	2234 (11.1)
Malignant tumor	734 (2.4)	53 (2.7)	270 (3.2)	411 (2.0)
Chronic pulmonary disease	6029 (19.8)	192 (9.8)	1303 (15.6)	4534 (22.52)
Chronic kidney disease	4953 (16.2)	153 (7.8)	1304 (15.6)	3496 (17.4)
**Number of patients with at least one observation after 90 days**	**12,424**	**884**	**5269**	**6271**
Mean Time of PASC Observation, days (SD)	312.67 (136.67)	319.76 (151.43)	385.79 (172.48)	234.47 (87.14)
Median Time of PASC Observation, days (IQR)	–	305 (221–385)	376 (253–514)	235 (168–281)
Minimum and Maximum Time of PASC Observation, days	90–852	90–852	90–729	90–550

a Data are n (%), n/N (%), or median (IQR). The differing denominators indicate missing data. Within the category sex, the category “other” was designated at some hospitals, but the results were censored due to the number being low.

### Study design

This study is divided into two phases: Phase 1 focused on defining PASC sub-phenotypes with structured EHR data using distributed learning. Phase 2 used the derived definitions for meta-analysis to provide population prevalence estimates and evaluate the temporal distribution of each of the PASC sub-phenotypes.

#### Defining PASC sub-phenotypes with structured EHR data using distributed learning (Phase 1)

**Fig. 1:**
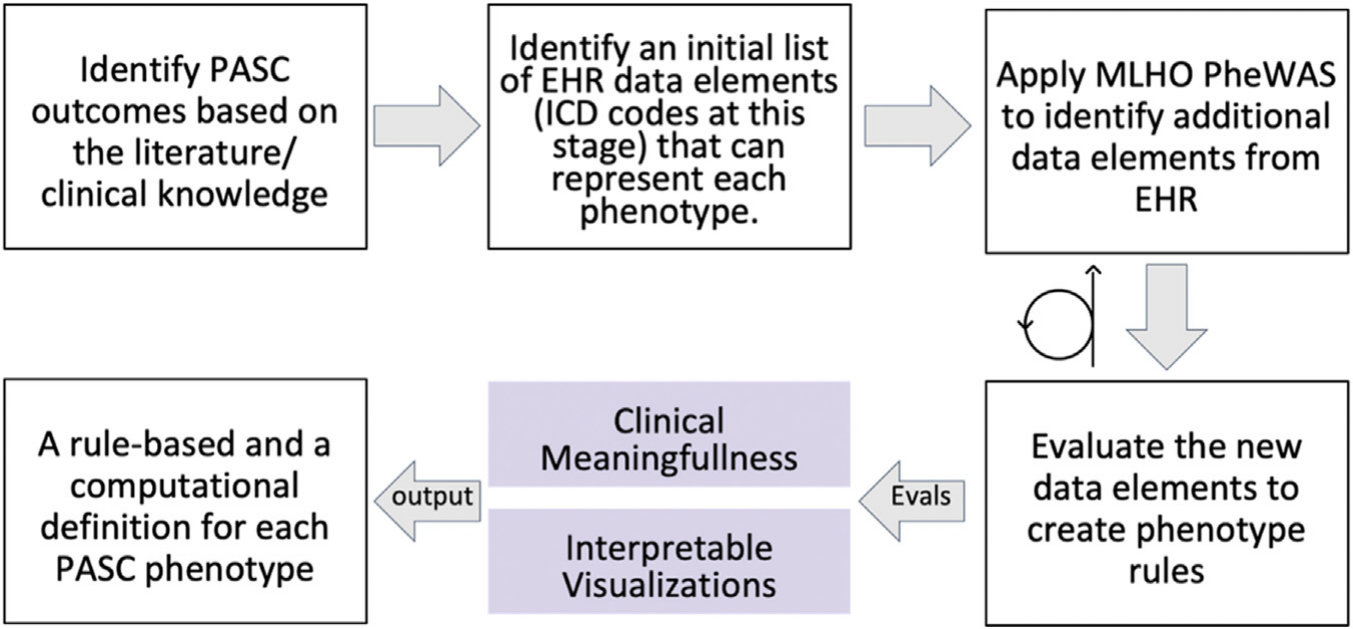
**Overview of the Deductive Study Pipeline in Phase 1 of the Study.** MLHO leverages the informatics infrastructures developed by the 4CE for a distributed study of PASC sub-phenotypes in a deductive data-driven pipeline, in which we augmented clinical knowledge using an iterative approach.

To define PASC sub-phenotypes, we applied a deductive approach in which we augmented clinical knowledge using an iterative data-driven approach (Fig. 1).

We employed clinical expertise to curate an initial list of structured data elements that would be available in the EHR that can then be used to define PASC sub-phenotypes, which we identified and validated in the clinical literature. PASC involves a number of symptoms that need to be identified through an electronic phenotyping algorithm that discovers EHR markers represented as ICD diagnosis and procedures, LOINC laboratory tests codes, and ATC and RxNorm medication codes required to characterize each specific sequela. The initial data elements were dubbed *core features* (see Appendix Glossary). The goal of compiling this initial list was to create a broad criterion for defining cohorts of patients with PASC sub-phenotypes. If a core feature was recorded in the medical record for the first time at 90 days or longer after the initial acute COVID-19 hospitalization, a patient record was flagged as possibly having a PASC sub-phenotype. The set of core features that serve as a starting point for labeling patients as having PASC is reported in Table S1).

The 4CE network has established procedures for distributed learning (through Docker) and has distributed queries for extracting EHR data in a specific data model with standard quality checks. To translate the 4CE data model and conform with its analytics distribution procedures, we developed a wrapper [https://github.com/rebeccamesa/pascPhen], an R package that implements the iterative association mining algorithm provided in Machine Learning for Health Outcome (MLHO) pipeline.^18^ The goal for the data-driven process was to augment the cohort definitions by identifying additional EHR data elements (see Appendix Glossary) that associate with the initial list and could potentially be used to enrich the clinical definition of PASC sub-phenotypes.

The MLHO algorithm uses a probabilistic approach to mining association rules. Association rule mining is an unsupervised technique that relies on frequency-based criteria for identifying co-occurrence patterns in large datasets. Association rule mining algorithms are designed for use with transactional data, such as data on consumer purchases or online browsing behavior. MLHO's entropy-based algorithm provides a powerful tool for capturing non-linear associations in clinical data while also incorporating sparsity in identifying associations.^20^

Phenome-wide association studies (PheWAS) identify associations between specific genetic variations and specific diseases or traits in a population. PheWAS studies analyze data from large EHR warehouses or biobanks to identify patterns of co-occurrence between different biomarkers and health outcomes. However, PheWAS association mining is based on p-values, which can be problematic when studying disease sub-phenotypes due to sensitivity to sample size. In contrast, MLHO uses an iterative feature selection algorithm that seeks to identify features that contain useful predictive information.^2^

**Fig. 2:**
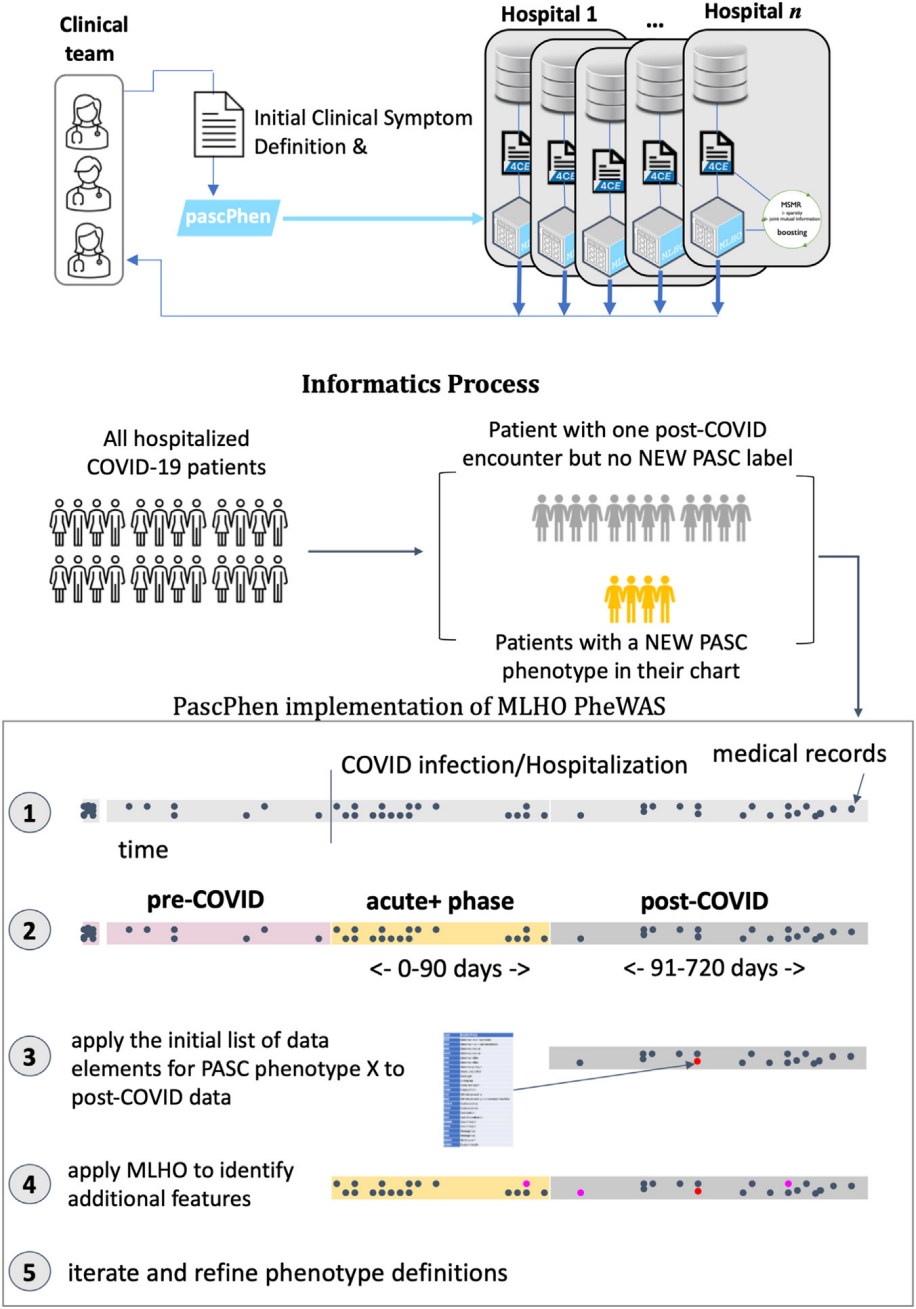
**The data-driven process for enriching initial PASC sub-phenotype definitions.** Leveraging the initial PASC sub-phenotype definitions, we developed a distributed representation learning that identifies additional EHR data elements (i.e., encounter records) that associate with a patient having a diagnosis code for a PASC problem 90 days or longer after COVID-19 hospitalization. The process included the following steps: 1. 4CE data model is transformed to MLHO input; 2. EHR data are time stamped based on the index data into pre-COVID, acute + phase, and post-COVID; 3. Using the initial data elements, we identified potential patients with specific symptoms after a SARS-2-CoV infection; 4. The initial (core) features are removed and MLHO is applied to identify data elements during the post-COVID and acute + phase that can predict the label for a given phenotype; 5. Step 4 is iterated 5 times to compute MLHO confidence score, which quantifies the number of times a feature is identified as a predictor for a prediction/classification task.

The MLHO PheWAS implementation in the PascPhen R package included the following steps (Fig. 2). First, the 4CE data model is transformed to the MLHO input data model and the index date (i.e., date of COVID-19 hospitalization) is set for all patients.

**Fig. 3:**
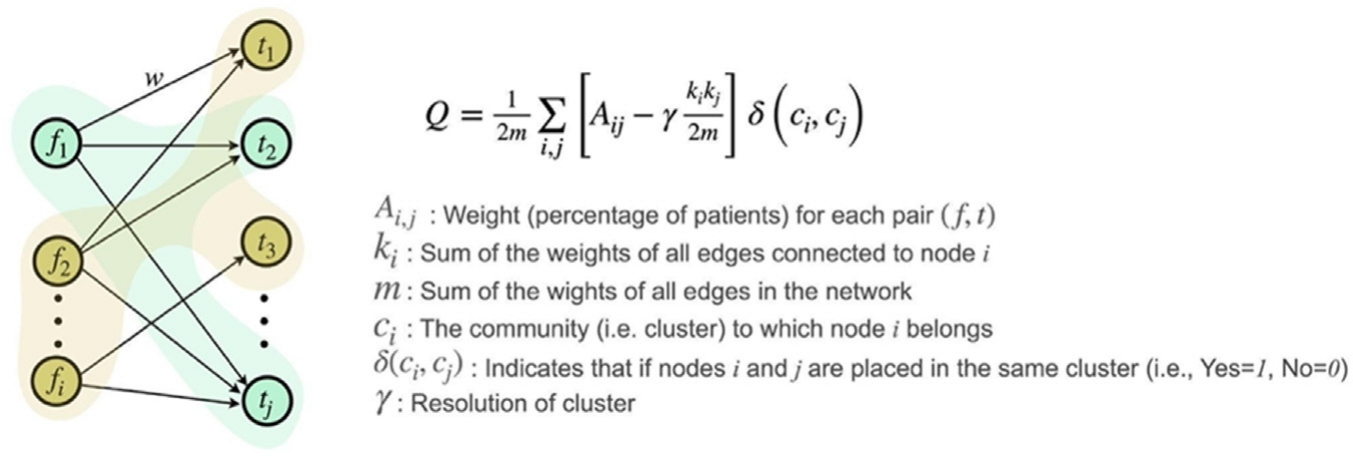
**Illustration of Louvain method used to cluster features.** This figure shows the graph structure used to cluster core and MLHO features. Nodes annotated with ***f*** represent the features, and ***t*** nodes show the time. The weight of each connection presents the percentage of patients diagnosed with corresponding feature ***f*** at time ***t***. In this example, clusters are separated using different colors.

**Fig. 4:**
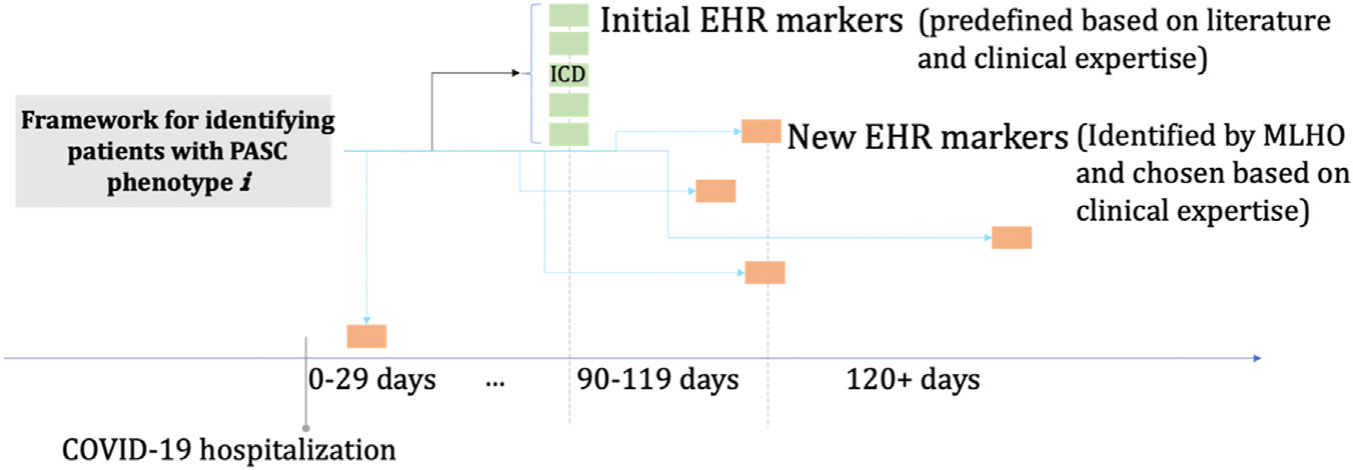
**Schematic construction of the augmented definition for a PASC sub-phenotypes.** An augmented definition for a PASC sub-phenotype encompassed time-stamped features from patients' EHRs. Core features (initial EHR markers) have an a priori temporal definition of being recorded for the first time 90 days or longer after the hospitalization. MLHO features (new EHR markers) can be observed any time post hospitalization, but are time stamped to capture the temporal relationships with the core features.

Second, EHR data are time-stamped based on the index date into pre-COVID (pre-hospitalization), acute + phase (0–90 days after hospitalization), and post-COVID (>90 days after hospitalization).

Third, using the initial core features, potential patients were identified with these specific symptoms after a SARS-2-CoV infection. A cohort is defined for each PASC sub-type if a core feature is present in a patient's medical record for the first time 90 days or longer since hospitalization. Patients are labeled as positive for a phenotype if they meet the minimum criteria definition (i.e., the presence of one core feature), negative if they do not meet the minimum criteria for the PASC sub-phenotype and have at least a follow up encounter during the post-COVID time. A one year look back period before the initial SARS-2-CoV infection was used for each patient.

Fourth, the core features are removed from the medical records and MLHO is applied to identify data elements during the post-COVID and acute-positive phase that can predict the label for a given phenotype. MLHO (a) uses the minimize sparsity, maximize relevance (MSMR) dimensionality reduction algorithm^21^^,^^22^ that leverages joint mutual information and sparsity screening to (b) train gradient boosting machine classification models (using the gbm package in R^23^) with 5-fold cross validation for (c) identifying EHR data elements that associate with a patient belonging to a given PASC sub-type cohort definition defined in step 3. Discrimination performance was measured by area under the receiver operating characteristic curve (AUROC) and reported with 95% confidence intervals.

Lastly, the fourth, previous, step is iterated five times to compute MLHO confidence score, which quantifies the number of times a feature is identified as a predictor for a prediction/classification task, considering possible discrepancies between the direction of the association (i.e., as a risk or protective factor). The idea is to iteratively apply this process until there are no more relevant clinical markers for redefining the phenotypes. The choice of the number of iterations was guided by computational resources available across different institutions. We also chose an odd number to facilitate federated analyses by using medians.

The PascPhen package performs as a wrapper and implements MLHO PheWAS on 4CE data extracts. The package produces flat file reports which we used for developing a visualization dashboard and evaluating clinical meaningfulness of the features identified by MLHO; henceforth, we call them *MLHO features*. This approach allowed the inclusion of temporal dimensions related to disease progression in the initial definition of PASC sub-phenotypes, by applying association rule mining to time-stamped features, as described above This way, one can discover new features that can be included in the new enriched definition of PASC sub-phenotypes.

The PascPhen package was distributed to five 4CE sites: four in the United States and one in Europe. Data was transmitted in aggregate. The package produced a unified report in HTML format from each site, including descriptive statistics on the patient population with core features, temporal distribution of the onset of each PASC sub-phenotype at the site, and new features identified through iterative MLHO applications that associated with the core features, with odds ratio, p-value, and 95% confidence intervals—Figure S2 presents a template report from phase 1 results.

MLHO features were validated by a team of eight clinicians who reviewed the features to assess if they were clinically meaningful and whether their incorporation into the original definition for each PASC sub-phenotype would result in an enrichment of the original definition. Since many of the PASC sub-phenotypes are symptoms or signs, any MHLO identified diseases, medications, laboratory values, and/or procedures that the team thought could explain that symptom or sign were included. Also, if there was a synonym of the PASC sub-phenotype, it would then be included. Further details of this selection process can be found in Strasser 2023 et al.^24^

The enriched definition was used to categorize patients into four distinct groups (see Appendix Glossary) based on the presence of MLHO and/or core features in their EHRs. Group 4 consisted of those patients with both the core and MLHO features at respective timelines. Group 3 included those only with the core features (90 or more days since hospitalization), and no MLHO features. Group 2 consisted of those only with the MLHO features, but not the core features. Finally, Group 1 lacked any of the core or augmented features (see Appendix Glossary).

#### Meta-analysis for PASC prevalence estimates and temporal distribution (Phase 2)

We applied the novel definitions of the PASC sub-phenotypes to the hospital systems for meta-analyses. Each site ran the PascPhen and returned harmonized summary statistics, which we exploited to estimate PASC prevalence, including the proportion of patients with (a) at least one PASC issue, (b) more than one PASC issue, and (c) each of the seven PASC sub-phenotypes.

We estimated the prevalence of overall PASC for MHLO and core symptoms, and the overlap between them, leveraging the Group 3 (individuals identified as PASC cases by core features) and Group 4 (individuals identified as PASC cases by core and MHLO features) definitions.

**Fig. 5:**
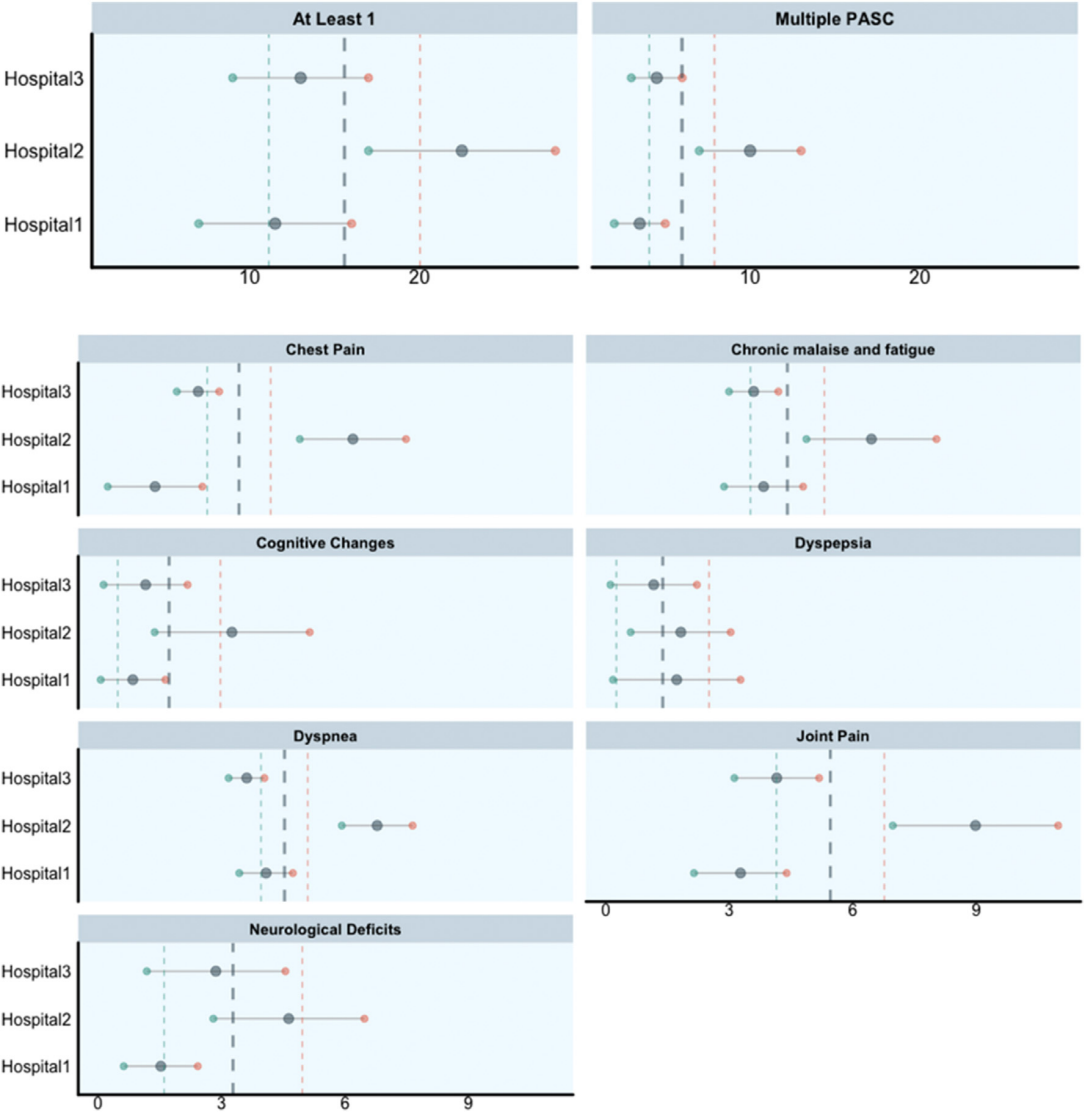
**Prevalence estimates for the overall PASC phenotype and specific PASC sub-phenotypes in the hospitalized population.** Each plot reports on the horizontal axes the prevalence values as percentages of subjects identified by CORE and/or MLHO features over the total of COVID-19 hospitalized subjects. Each row represents a site via lollipop plots, reporting lower limit (green, col1), upper limit (red, col2) and average (gray, col3) values. Vertical lines represent average prevalence across hospitals, using as weight the number of subjects enrolled in the analyses by each site.

To estimate the prevalence ranges, we defined the lower limit as the number of subjects identified by both MHLO and core features during the post-COVID time window (Group 4) divided by the population during the same period. We defined the upper limit as the number of subjects identified only by core features during the post-COVID time window (Group 3) plus the number of subjects identified by both MHLO and core features (Group 4), divided by the population during the same period. We then calculated their average. The reported prevalence is the average of the lower and upper limits. Results (Section 3.3. and Fig. 5) report the values as Prevalence Average (Lower Limit–Upper Limit).

For each hospital, we computed the overall PASC prevalence among all the hospitalized cases, including data from patients with at least one or more of the PASC symptoms. The same estimates were calculated for single PASC sub-phenotypes. Prevalence estimates and the following analyses were performed on the most prevalent PASC sub-phenotypes. We computed weighted average prevalence across hospitals, weighting by the number of subjects enrolled in the analyses by each site.

We studied the temporal distribution of the most prevalent PASC sub-phenotypes. We compared the overall count of features, in 10-day time windows beginning 90 days after hospitalization. We described the distributions as raw data (i.e., features count in time windows) and via kernel density estimates. We performed a Wilcoxon rank sum test, with continuity correction and p-value Bonferroni adjustment, to compare the features’ occurrence in time. We performed an analysis within each hospital, to account for potential confounding factors due to center-effect clustering.

To cluster MLHO and core features linked by time of presentation for a feature and number of patient who had a given feature at a given time, we used the Louvain community detection algorithm.^25^ This approach leverages multi-level optimization to maximize the modularity score (*Q*) of each cluster, where modularity ranges from −1 to 1 and measures the density of links within clusters versus links between clusters. We used core and MLHO features and temporal data to define the nodes of the input graph to the Louvain method and added the percentage of patients for each pair (feature, time) as a weight to the corresponding connections (See Fig. 3). This will allow the Louvain approach to leverage its flexibility in detecting similar sub-communities and incorporate more granular temporal variables in defining the clusters rather than clustering the features based on their overall patient/time distribution. The resolution of clusters is defined by γ, an algorithmic parameter to define the granularity of clusters. Lower values of γ result in fewer (and larger) clusters. To remain consistent with the original definition of modularity and considering the small size of our graphs, we set γ = 1. Similar to other unsupervised learning approaches, the choice of the optimal number of clusters might impact the interpretation of the results. However, as the Louvain method only uses the pre-defined number of clusters (resolution) to determine the granularity of clusters, this parameter does not affect the plausibility of our interpretations.

### Ethics

The use of EHR data at each institution was approved by local Institutional Review Boards with waiver of patient consent. Certifications of IRB waivers or approval were collected by the 4CE consortium. This study was determined to be exempt as secondary research by further institutional review boards at Bordeaux University Hospital, Harvard Medical School, Massachusetts General Hospital, Northwestern University, University of Kentucky, University of Pittsburgh.

### Role of funding source

The funders have no role in data collection, analysis, interpretation, writing of the manuscript and the decision to submit the manuscript.

## Results

### Characteristics of the study population

The study population consisted of a total of 30,422 hospitalized COVID-19 patients from three hospital systems who tested positive for SARS-CoV-2 between the first quarter of 2020 (2020-Q1) through the first quarter of 2022 (2022-Q1). Of these subjects 12,424 had at least one observation after 90 days from the first positive test. Table 1 presents the overall characteristics of the study population, including comorbidities at baseline, and summary statistics on PASC features.

### PASC sub-phenotypes definition

We retained in the analyses the phenotypes that were identified by at least two of the three hospital systems. We provide an augmented definition for seven PASC sub-phenotypes using structured EHR data: Joint pain, Dyspnea, Chronic malaise and fatigue, Chest pain, Neurological deficits, Dyspepsia, and Cognitive changes. The list of EHR data elements to construct initial cohorts of PASC sub-phenotypes is provided in Table S1 (Appendix).

Table 2 presents the AUROC obtained from each site for each of the PASC sub-phenotypes. Most of the sub-phenotypes were above an acceptable AUROC threshold (>0.8). That is, sub-phenotypes, developed at each site, were able to identify new features from the EHRs that classified patients who had a PASC core feature for the first time 90 days, or longer post hospitalization, with acceptable discrimination accuracy, when the core features were removed from the data.

**Table 2:** Discrimination performance (measured by AUROC) for each PASC sub-phenotype across the 3 hospital systems.

PASC sub-phenotype	Hospital system 1	Hospital system 2	Hospital system 3
Joint Pain	0.78 [0.77–0.80]^a^	0.84 [0.84–0.84]	0.82 [0.82–0.83]
Dyspepsia	0.81 [0.80–0.82]	0.82 [0.81–0.82]	0.80 [0.80–0.81]
Chronic malaise and fatigue	0.90 [0.89–0.91]	0.85 [0.85–0.86]	0.84 [0.84–0.84]
Dyspnea	0.91 [0.89–0.91]	0.81 [0.81–0.82]	0.86 [0.85–0.86]
Cognitive Changes	0.86 [0.85–0.87]	0.87 [ 0.87–0.88]	0.87 [0.86–0.87]
Neurological deficits	0.77 [0.76–0.78]	0.84 [0.84–0.84]	0.85 [0.85–0.85]
Chest pain	–	0.88 [0.87–0.88]	0.87 [0.87–0.88]

a 95% Confidence Intervals. The table shows the area under the receiver operating characteristics curve obtained from each site running MLHO on the subset of their EHR data without core features. The classification task was to build a model that can classify the patients who had at least a core feature for a PASC sub-phenotype by using their EHR data from after COVID-19 infection. Core features were removed and remaining features were time-stamped.

The clinical team categorized the features identified by MHLO based on the underlying relationship between the identified data element and phenotypes. Four types of relationships were identified as potentially describing the association of the MHLO feature to the phenotype. First, a disease may have been identified by MHLO if the disease's primary symptom matched that of the phenotype. For example, in the case of “joint pain”, a new diagnosis of “bilateral primary osteoarthritis of the knee” is likely representative of the “joint pain” phenotype. The second relationship identified was a laboratory test order that suggested the phenotype. For example, in the case of “chronic fatigue”, ordering “ferritin” could imply the ordering provider was trying to understand the underlying etiology of a patient's anemia, which often presents as fatigue. Thus, ordering ferritin implies the patient may have fatigue. The third relationship was a procedure that suggests the underlying phenotype. For example, a CT scan of the chest may be related to a clinical provider trying to determine the etiology of dyspnea. Finally, in some cases elements were identified that were near synonyms to the original definition, such as hypoxemia representing dyspnea, but had been missed in the original definition. Table 3 shows a sample of the features identified for each phenotype and their relationship to that phenotype. For a complete list of features used to describe each phenotype see Tables S2 and S3 and Figure S3. The features identified by MHLO, and that had a medical rationale explained by one of the four categories, were then incorporated into the new augmented definition.

**Table 3:** Sample of the augmented features identified by MLHO that are suggestive of dyspnea, chronic fatigue, and joint pain.

	PASC subtype		
	Dyspnea	Chronic fatigue	Joint pain
Core features	R06^a^–Abnormalities in Breathing	F53^a^–Fatigue	M25.5^a^ Pain in Joint
Sample augmented features			
Diseases with related symptom	I48.0–Paroxysmal Atrial Fibrillation	E03.9–Hypothyroidism	M17.0–Bilateral primary osteoarthritis of the knee
Laboratory tests suggestive of symptom	LNC 38065–7 D-dimer	LNC 2276–4 Ferritin	LNC 1988–5 CRP
Procedures suggestive of symptom	CAT Chest	–	–
Near synonym	R09.02–Hypoxemia	–	M54.5–Low Back Pain

a Includes all ICD codes within the parental group.

The new augmented definition consists of the core features plus the time-stamped MLHO features (Fig. 4). Based on the presence of core and MLHO features, we classified patients into four groups: (1) those who do not have any of these features in their EHRs and thus are very unlikely to have PASC, (2) those who only have one of more MLHO features who may have the PASC sub-phenotype, but not likely, (3) patients who have a core feature in their EHR for the first time 90 days or longer post-hospitalization, whom we consider likely to have the PASC sub-phenotype, and (4) those who have both core and MLHO features, who are very likely to suffer from the given PASC sub-phenotype. The signal detection schema is illustrated in Figure S4 for the PASC sub-phenotype joint pain.

### PASC prevalence estimate

We found that on average 15.7 (Lower Limit 11.12—Upper Limit 20.03) percent of the hospitalized COVID-19 patients had at least one PASC problem and 5.98 (Lower Limit 4.06–Upper Limit 7.91) percent of the hospitalized COVID-19 patients had multiple problems (see Fig. 5 and Tables S4 and S5). When the PASC sub-phenotypes were examined separately, the most prevalent were Joint pain and Dyspnea, with an average prevalence of 5.45 (Lower Limit 4.14–Upper Limit 6.76) and 4.53 (Lower Limit 3.95–Upper Limit 5.09), respectively. Dyspepsia and Cognitive changes were the least common (less than two percent on average).

### PASC symptoms temporal patterns

**Fig. 6:**
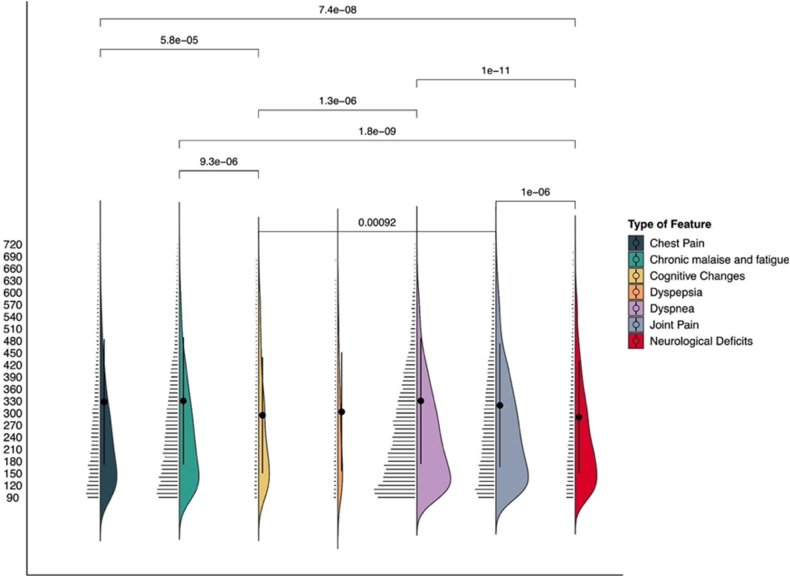
**PASC sub-phenotype features temporal distribution.** For each PASC sub-phenotype we report the number of features in each 30-day time window. The plot, which reports days on the y-axis, illustrates kernel densities on the right of each PASC sub-phenotype, mean and standard deviation (the points and the intervals over the violin plots), and jittered raw data points on the left. Temporal distributions of PASC features were compared by pairwise Wilcoxon test, with a Bonferroni correction; p-values for significant results (<0.05) are reported on the vertical lines that connect different PASC sub-phenotypes.

We analyzed the temporal distribution of the most prevalent PASC sub-phenotypes (i.e., prevalence greater than 2%), excluding Cognitive changes and Dyspepsia. Chronic malaise and Fatigue and Dyspnea symptoms appear later (i.e., on average the features are detected 330 days after the primary infection) while Neurological deficits appear earlier (on average 289 days after the primary infection). Fig. 6 illustrates the distribution and the temporal comparison of PASC sub-phenotypes. The only PASC sub-phenotype found to have a statistically different distribution is Neurological deficits, with symptoms appearing earlier (on average, by about a month) than the other PASC sub-phenotypes. The distribution comparisons are reported in Tables S6, and for each site in Tables S7, S8 and S9. The features distribution over time is reported in Tables S10 and S11.

**Fig. 7:**
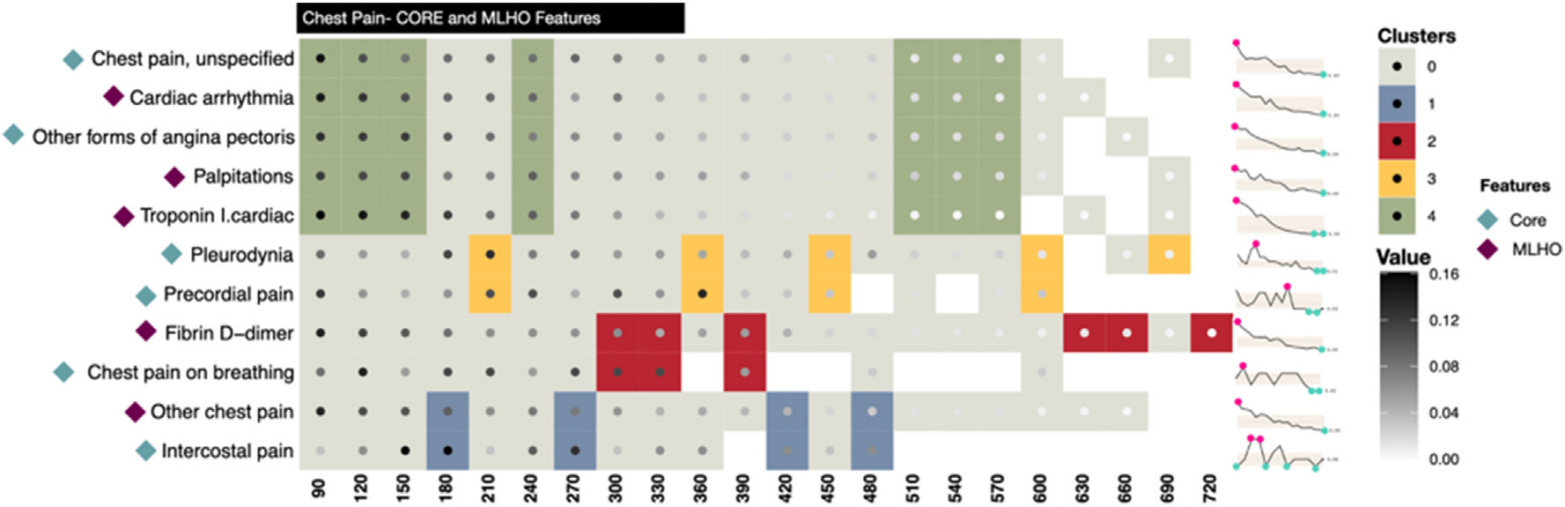
**Clustered presentation and temporal distribution of the core and MLHO features.** The clusters are defined using Louvain clustering. Each node of the clustering graph is presented as ***(f,t,p)***, where ***f*** presents the feature, ***t*** presents the time and ***p*** shows the percentage of patients. Blank squares present missing values and the gradient-colored dots show the value of ***p***. The diamonds next to the features on the y-axis define the type of each feature (i.e., core vs. MLHO) and the sparklines on the right side present the overall temporal distribution of each feature.

Fig. 7 illustrates the temporal distribution of each feature classified under Chest pain and recorded during the post-COVID period (i.e.,> 90 days after COVID-19 hospitalization). Features are clustered on the basis of their temporal distribution. The colored squares show the results of Louvain clustering,^25^ which was used to detect features with similar temporal distributions. The prevalence of PASC sub-phenotypes is illustrated using gradient-colored points for each pair <*f*, *t*>, where *f* represents features and *t* time. The sparklines on the right side of the figure show the overall temporal trend of the prevalence of each feature, with maximum and minimum values annotated in red and blue dots, respectively. Evaluating the granular, temporal distribution of each of the sub-phenotypes can be useful for further understanding of common sequential patterns. In the case of chest pain (Fig. 7) the earliest components that make up the sub-phenotype are “cardiac arrhythmia”, “palpitations”, “chest pain, unspecified”, and “troponin”. These all point to a cardiac cause rather than a muscular, pulmonary, or neuropathic one, whereas the later clusters include features like “pleurodynia”, “intercostal pain”, “d-dimer”, and “chest pain on breathing”. These later components point to a pulmonary origin for the chest pain. The supplement contains the Louvain clustering results for each of the sub-phenotypes (See Figures S5, S6, S7 and S8).

## Discussion

PASC has evolving properties that need to be defined in a timely manner to mitigate health, social, and economic impacts. Characterizing and understanding sub-types of PASC can lead to better outcomes for individuals affected by the condition and improve its overall understanding and management. While the potential pitfalls in the use of real-world data for studying PASC are many, structured EHR data offer feasible and low-cost solutions for establishing screening procedures for PASC's multiple existing and future sub-phenotypes. The results of this study can be used to provide reliable screening capacities for PASC sub-phenotypes.

We created a high-throughput approach to characterizing PASC sub-phenotypes from structured EHR data. It is high-throughput because the definitions are simple to implement and rely only on structured EHRs that are widely available. This study also provides one of the most granular characterizations of PASC sub-phenotypes to date. Evaluating the granular, temporal distribution of each phenotype can reveal more PASC sub-phenotypes and more information about PASC in general. Characterizing the sub-phenotypes of PASC can guide assessment and diagnosis of the post-COVID-19 problems, and improve treatment and management plans.

To date, the definition of PASC is highly variable, with definitions comprising the presence of persistent new-onset symptoms ranging from 30 to 90 days post-SARS-CoV-2 infection.^9^ However, the natural history of PASC remains poorly understood. Our analyses suggest that there is a substantial population of patients with persistent symptoms that present more than 180 days post-SARS-CoV-2 infection, associated with specific laboratory data longitudinally across all seven PASC sub-phenotypes. This suggests that the time-based criteria of PASC could potentially be more stringent to increase the specificity of the diagnosis of PASC. Characterizing PASC sub-phenotypes will also enable the identification of at-risk populations.

Based on data from multiple institutions, we estimated that approximately 15 percent (11%–20%) of hospitalized COVID-19 patients will have at least one PASC complication and approximately five percent (4%–8%) will have multiple problems. Our findings are generally consistent with prior estimates of prevalence, which show that approximately between 13 and 19 ^26^^,^^27^ percent of patients with a previous COVID-19 infection report symptom of PASC.

However, prevalence estimates also vary widely across studies, owing to differences in PASC definitions and methodologies to measure the outcome. O'Mahoney et al., in a systematic review of 48 studies - although based on smaller populations (at least 100 people) and including self-reported COVID-19 symptoms at ≥28 days following infection onset - concluded that approximately 52.6% (95% CI 43.5%–61.6%) of hospitalized COVID-19 survivors experienced at least one symptom.^28^

While the prevalence of PASC is still being debated, we were able to provide prevalence estimates for seven PASC sub-phenotypes, with Joint pain and Dyspnea having the highest prevalence at 5.45 (4.14–6.76) and 4.53 (3.95–5.09), respectively. Estimating the prevalence of PASC by sub-phenotype can aid in the development of effective interventions and support for PASC patients. Healthcare providers and public health officials can leverage these estimates to better understand the overall burden of PASC in the population and allocate resources accordingly.

The Louvain clustering technique demonstrates how the specific underlying features that define a phenotype are organized in time and related to one another. This analysis can provide further insight into the underlying etiology of a symptom and how that symptom's signal changes over time. For example, in the case of chest pain, the early signal detected is related to a cardiac etiology rather than a pulmonary one. A number of studies have previously described cardiac manifestations of acute COVID-19 including myocarditis, stress cardiomyopathy, and myocardial infarction.^29^^,^^30^ This early signal detected by our algorithm could be related to the cardiac manifestations of acute COVID-19, which were not detected in the EHR until shortly after the acute period. Our algorithm suggests that the late chest pain signal has a different etiology and is no longer associated with an underlying cardiac diagnosis.

There are several limitations to our work, derived from the lack of patient-level data for the meta analyses, which will be the focus of our subsequent analyses, and the inherent features of structured EHR data. We only evaluated those PASC sub-phenotypes for which we had a sufficient number of patients based on the initial definition in the institutional EHR data. Some PASC syndromes such as chronic fatigue may not be completely captured in EHRs. Fatigue has been frequently reported as a sequel of COVID-19 in the literature among non-hospitalized COVID-19 patients (∼35 percent prevalence^28^). This could have resulted in an underestimation of patients afflicted with at least one PASC sub-phenotype, which could explain our lower prevalence estimates when compared with other studies.

Due to the pre-specified 4CE data extraction schema, we did not have access to all EHR data elements in local data extracts. Therefore, our data-driven methodology may have overlooked additional data elements that could have been useful for constructing the augmented PASC definitions. As with any observational study that uses existing EHR data of hospitalized patients, it is likely that unmeasured confounders could affect the associations we have found. Furthermore, hospitalization also introduces the wider biases associated with hospitalization in many settings such as race and deprivation.

Of particular interest, but beyond the scope of this study, is the possible effect of unmeasured mediators, such as hospital characteristics, health and social policies, social structure and capital, and built environment attributes. These features could, and likely do, influence the prevalence of PASC sub-phenotypes in specific demographic groups and/or geographical regions over time. Numerous methods exist for adjusting for unmeasured confounding, and future studies of PASC, especially those investigating the temporal subtyping of PASC, should include these methods, in addition to rigorous causal analysis.

Characterizing PASC sub-phenotypes via EHR is critical for managing the COVID-19 pandemic side effects in the coming years, but also complicated by several factors, including varying quality of EHR data owing to lack of documentation of symptoms and signs that may be associated with PASC. PASC occurs in a temporal context, with changes in its sub-phenotypes possibly resulting from differences in disease severity, the appearance or disappearance of certain symptoms (such as fatigue or dyspnea) over time, and the effects of treatments on the course of acute COVID. Our machine learning approach to characterizing PASC sub-phenotypes can help researchers identify cohorts for further study and enable clinicians to recognize evolving PASC sub-phenotypes while providing patient care.

## Contributors

**Design and Conceptualization of the study:** Arianna Dagliati, Zachary H. Strasser, Zahra Shakeri Hossein Abad, Rebecca Mesa, Gilbert S. Omenn, John H. Holmes, Shawn N. Murphy, Hossein Estiri.

**Data Collection:** Riccardo Bellazzi, Darren W. Henderson, Jeffrey G. Klann, Yuan Luo, Michele Morris, Malarkodi Jebathilagam Samayamuthu, Guillaume Verdy, Zongqi Xia, Hossein Estiri.

**Data Analysis or Interpretation**:

Arianna Dagliati, Zachary H Strasser, Zahra Shakeri Hossein Abad, Darren W Henderson, Jeffrey G Klann, Yuan Luo, Rebecca Mesa, Bryce W.Q.Tan, Shyam Visweswaran, Kavishwar B Wagholikar, Zongqi Xia, John H Holmes, Shawn N Murphy, Hossein Estiri.

**Drafting and Revision of Manuscript**:

Arianna Dagliati, Zachary H Strasser, Zahra Shakeri Hossein Abad, Riccardo Bellazzi, Darren W Henderson, Jeffrey G Klann, Yuan Luo, Michele Morris, Gilbert S. Omenn, Malarkodi Jebathilagam Samayamuthu, Bryce W.Q. Tan, Guillaume Verdy, Shyam Visweswaran, Kavishwar B Wagholikar, Zongqi Xia, John H Holmes, Shawn N Murphy, Hossein Estiri.

**Underlying Data Verification**:

Zachary H Strasser, Darren W Henderson, Jeffrey G Klann, Yuan Luo, Michele Morris, Guillaume Verdy, Hossein Estiri, 4CE Conortium.

All authors read and approved the final version of the manuscript.

## Data sharing statement

The analytic code is made available on GitHub https://github.com/rebeccamesa/pascPhen. The full patient-level dataset contains sensitive and potentially re-identifiable data. Therefore, it cannot currently be made available directly. Data may be made available to affiliated researchers given the MGB IRB approval. For access to aggregated data, please email 4ce@transmartfoundation.org. Baseline data can be found and downloaded at www.covidclinical.net.

## Declaration of interests

Riccardo Bellazzi is shareholder of Biomeris s. r.l. Gilbert Omenn holds patents for U.S. Application No. 16/169,048 Filed: 24-October- 2018 and License 2023–0632 with Radial Therapeutics, Inc.; Invention Disclosure No. 2022-382.

## Appendix A Supplementary data

APPENDIX

4ceMembers
