# Supplementary material for: Characterization of long COVID temporal sub-phenotypes by distributed representation learning from electronic health record data: a cohort study
Source: eClinicalMedicine. 2023 Sep 14;64:102210. doi: 10.1016/j.eclinm.2023.102210 (PMC10511779; doi:10.1016/j.eclinm.2023.102210)
Supplement: APPENDIX [file mmc1.docx]

**Appendix**

**Glossary**

**EHR data elements** are the components of encounter records, that is all the codes (ICD diagnosis codes, ATC treatments codes and LOINC laboratory test codes specified by the 4CE format) collected in the observation period form EHR data.

**Core features** are contained in the clinical/expert-curated list of EHR data elements that can broadly define a PASC subtype (table S1 in the Appendix)

**MLHO features** are EHR data elements identified by the ML pipeline that associate with the core features

**Augmented features** are the subset of MHLO features that were selected by clinical experts as meaningful (see supplementary tables S2 and S3)

**Sub-phenotypes** are possible clinical manifestations of long COVID that have been previously identified in the literature. A patient can have none or more than one sub-phenotype. **PASC sub-phenotypes** define a homogenous group of subjects identified by the core features and then refined by the augmented features. We initially considered the PASC subtypes listed with the core features in table S1. This study then focused on the seven PASC subtypes refined by the augmented features for further study.

**Groups** indicate patient’s status under a PASC subtype based on the type of features: (1) did not have any of the core or augmented features, (2) patient only has the core features, (3) patient only has the augmented feature, and (4) patient has both core and augmented feature.

**Clusters** refer to subgroups of features with a similar onset. It’s important to note that this clustering analysis was done as a meta-analysis on harmonized summary statistics and not at patient level.

**Distributed Representation Learning** in this study refers to process of learning EHR features (i.e., **Representations**) for defining PASC subtypes from multiple institutions by distributing the ML algorithm. In this context, the task of distributing the ML algorithm (i.e. **Distributed machine learning**) refers to the practice of training the same models (distributed via R scripts and Dockers) across multiple devices based in the different institutions. Thus, the models are trained locally on local data sets formatted accordingly 4CE format, then each institution shares its findings as aggregated results.

**ML pipeline** refers to a set of procedures that applies machine learning and associated methods that are applied sequentially to accomplish a task such as classification or prediction.

**Deductive Pipeline** refers to the sequential process we applied in this study for making inference about PASC definitions by going from general rules to specific conclusions


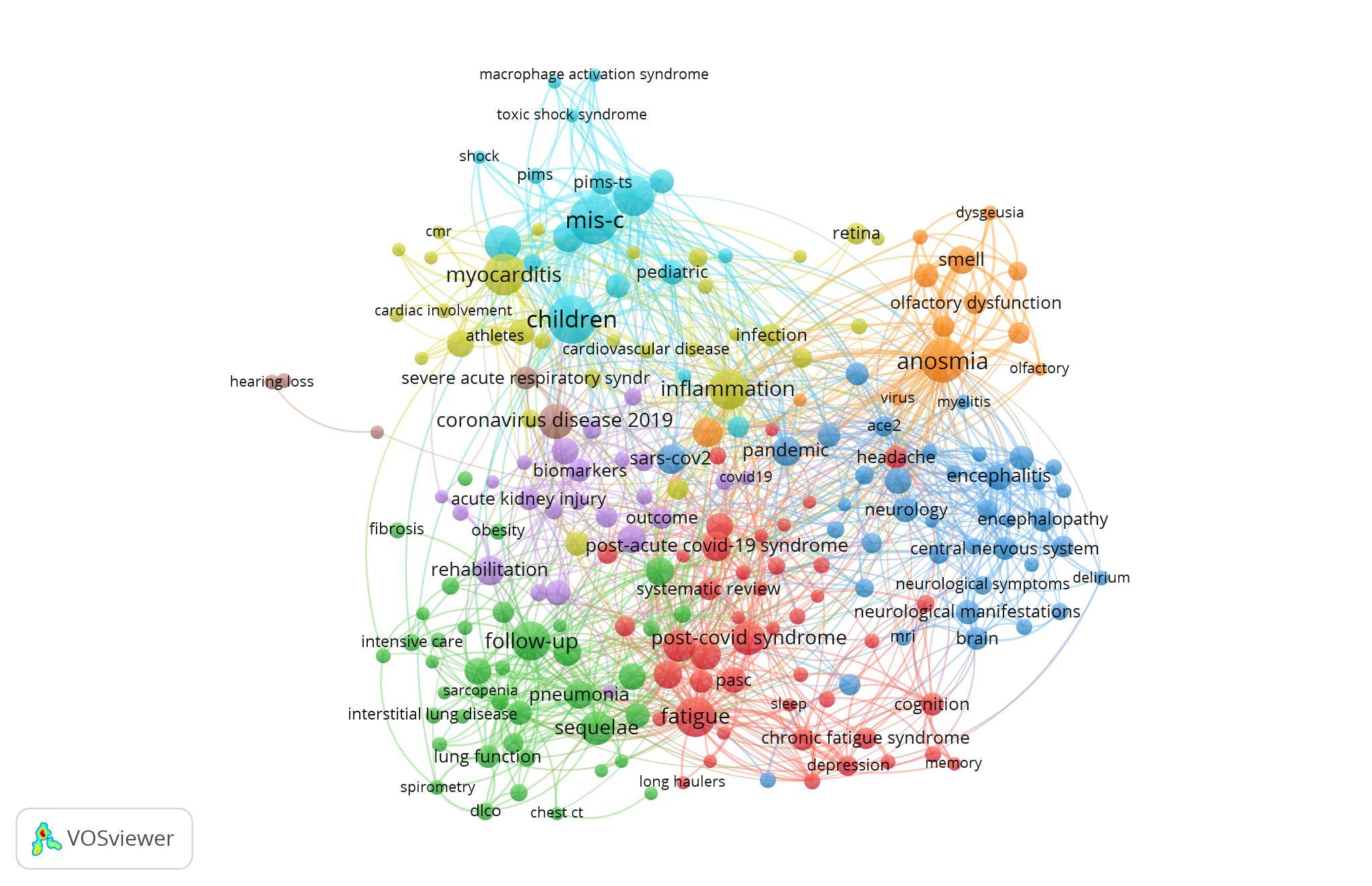


**Figure S1. Literature overview, keywords occurrence obtained via VOSViewer.**

**Table S1. Initial list of PASC subtypes with their associated core definition**

| **PASC Subtype** | **Code** | **Description** |
| --- | --- | --- |
| **Anxiety disorder** | F410 | Panic disorder [episodic paroxysmal anxiety] without agoraphobia |
|  | F411 | Generalized anxiety disorder |
|  | F413 | Other mixed anxiety disorders |
|  | F418 | Other specified anxiety disorders |
|  | F419 | Anxiety disorder, unspecified |
| **Brain fog** | R419 | Unspecified symptoms and signs involving cognitive functions and awareness |
| **Chest pain** | R071 | Chest pain on breathing |
|  | R072 | Precordial pain |
|  | R0781 | Pleurodynia |
|  | R0782 | Intercostal pain |
|  | R0789 | Other chest pain |
|  | R079 | Chest pain, unspecified |
| **Chronic malaise and fatigue** | R530 | Neoplastic (malignant) related fatigue |
|  | R531 | Weakness |
|  | R532 | Functional quadriplegia |
|  | R5381 | Other malaise |
|  | R5382 | Chronic fatigue, unspecified |
|  | R5383 | Other fatigue |
| **Cognitive changes** | R410 | Disorientation, unspecified |
|  | R411 | Anterograde amnesia |
|  | R412 | Retrograde amnesia |
|  | R413 | Other amnesia |
|  | R414 | Neurologic neglect syndrome |
|  | R4181 | Age-related cognitive decline |
|  | R4182 | Altered mental status, unspecified |
|  | R4183 | Borderline intellectual functioning |
|  | R41840 | Attention and concentration deficit |
|  | R41841 | Cognitive communication deficit |
|  | R41842 | Visuospatial deficit |
|  | R41843 | Psychomotor deficit |
|  | R41844 | Frontal lobe and executive function deficit |
|  | R4189 | Other symptoms and signs involving cognitive functions and awareness |
|  | R419 | Unspecified symptoms and signs involving cognitive functions and awareness |
| **Decreased estimated glomerular filtration rate** | N182 | Chronic kidney disease, stage 2 (mild) |
|  | N183 | Chronic kidney disease, stage 3 (moderate) |
|  | N1830 | Chronic kidney disease, stage 3 unspecified |
|  | N1831 | Chronic kidney disease, stage 3a |
|  | N1832 | Chronic kidney disease, stage 3b |
|  | N184 | Chronic kidney disease, stage 4 (severe) |
| **Dementia** | F0280 | Dementia in other diseases classified elsewhere without behavioral disturbance |
|  | F0281 | Dementia in other diseases classified elsewhere with behavioral disturbance |
|  | F0390 | Unspecified dementia without behavioral disturbance |
|  | F0391 | Unspecified dementia with behavioral disturbance |
| **Depressive symptoms** | F320 | Major depressive disorder, single episode, mild |
|  | F321 | Major depressive disorder, single episode, moderate |
|  | F322 | Major depressive disorder, single episode, severe without psychotic features |
|  | F323 | Major depressive disorder, single episode, severe with psychotic features |
|  | F324 | Major depressive disorder, single episode, in partial remission |
|  | F325 | Major depressive disorder, single episode, in full remission |
|  | F3281 | Premenstrual dysphoric disorder |
|  | F3289 | Single episode of 'masked' depression, not otherwise specified |
|  | F329 | Major depressive disorder, single episode, unspecified |
|  | F330 | Major depressive disorder, recurrent, mild |
|  | F331 | Major depressive disorder, recurrent, moderate |
|  | F332 | Major depressive disorder, recurrent severe without psychotic features |
|  | F333 | Major depressive disorder, recurrent, severe with psychotic symptoms |
|  | F3340 | Major depressive disorder, recurrent, in remission, unspecified |
|  | F3341 | Major depressive disorder, recurrent, in partial remission |
|  | F3342 | Major depressive disorder, recurrent, in full remission |
|  | F338 | Other recurrent depressive disorders |
|  | F339 | Major depressive disorder, recurrent, unspecified |
| **Diffuse myalgia** | M791 | Myalgia |
| **Dyspepsia** | K30 | Functional dyspepsia |
|  | K210 | Gastro-esophageal reflux disease with esophagitis |
|  | K219 | Gastro-esophageal reflux disease without esophagitis |
| **Dyspnea** | R0600 | Dyspnea, unspecified |
|  | R0601 | Orthopnea |
|  | R0602 | Shortness of Breath |
|  | R0603 | Acute respiratory distress |
|  | R0609 | Other forms of dyspnea |
| **Fibrotic changes on CT scans of the chest** | J8410 | Pulmonary fibrosis, unspecified |
| **Flare of autoimmune disease** | D899 | Disorder involving the immune mechanism, unspecified |
| **Graves’ disease** | E0500 | Thyrotoxicosis with diffuse goiter without thyrotoxic crisis or storm |
|  | E0501 | Thyrotoxicosis with diffuse goiter with thyrotoxic crisis or storm |
| **Hair loss** | L630 | Alopecia (capitis) totalis |
|  | L631 | Alopecia universalis |
|  | L632 | Ophiasis |
|  | L638 | Other alopecia areata |
|  | L639 | Alopecia areata, unspecified |
| **Hasimoto’s thyroiditis** | E063 | Autoimmune thyroiditis |
| **Headaches** | R51 | Headache |
| **Increased rate of diabetic ketoacidosis** | E1010 | Type 1 diabetes mellitus with ketoacidosis without coma |
|  | E1011 | Type 1 diabetes mellitus with ketoacidosis with coma |
|  | E1110 | Type 2 diabetes mellitus with ketoacidosis without coma |
|  | E1111 | Type 2 diabetes mellitus with ketoacidosis with coma |
|  | E1310 | Other specified diabetes mellitus with ketoacidosis without coma |
|  | E1311 | Other specified diabetes mellitus with ketoacidosis with coma |
|  | E0910 | Drug or chemical induced diabetes mellitus with ketoacidosis without coma |
|  | E0911 | Drug or chemical induced diabetes mellitus with ketoacidosis with coma |
|  | E0810 | Diabetes mellitus due to underlying condition with ketoacidosis without coma |
|  | E0811 | Diabetes mellitus due to underlying condition with ketoacidosis with coma |
| **Irritable bowel syndrome** | K580 | Irritable bowel syndrome with diarrhea |
|  | K581 | Irritable bowel syndrome with constipation |
|  | K582 | Mixed irritable bowel syndrome |
|  | K588 | Other irritable bowel syndrome |
|  | K589 | Irritable bowel syndrome without diarrhea |
| **Joint pain** | M2550 | Pain in unspecified joint |
|  | M25511 | Pain in right shoulder |
|  | M25512 | Pain in left shoulder |
|  | M25519 | Pain in unspecified shoulder |
|  | M25521 | Pain in right elbow |
|  | M25522 | Pain in left elbow |
|  | M25529 | Pain in unspecified elbow |
|  | M25531 | Pain in right wrist |
|  | M25532 | Pain in left wrist |
|  | M25539 | Pain in unspecified wrist |
|  | M25541 | Pain in joints of right hand |
|  | M25542 | Pain in joints of left hand |
|  | M25549 | Pain in joints of unspecified hand |
|  | M25551 | Pain in right hip |
|  | M25552 | Pain in left hip |
|  | M25559 | Pain in unspecified hip |
|  | M25561 | Pain in right knee |
|  | M25562 | Pain in left knee |
|  | M25569 | Pain in unspecified knee |
|  | M25571 | Pain in right ankle and joints of right foot |
|  | M25572 | Pain in left ankle and joints of left foot |
|  | M25579 | Pain in unspecified ankle and joints of unspecified foot |
| **Loss of taste and smell** | R430 | Anosmia |
|  | R431 | Parosmia |
|  | R432 | Parageusia |
|  | R438 | Other disturbances of smell and taste |
|  | R439 | Unspecified disturbances of smell and taste |
| **Lung transplantation** | Z942 | Lung transplant status |
| **Mood disorders** | F3010 | Manic episode without psychotic symptoms, unspecified |
|  | F3011 | Manic episode without psychotic symptoms, mild |
|  | F3012 | Manic episode without psychotic symptoms, moderate |
|  | F3013 | Manic episode, severe, without psychotic symptoms |
|  | F302 | Manic episode, severe with psychotic symptoms |
|  | F303 | Manic episode in partial remission |
|  | F304 | Manic episode in full remission |
|  | F308 | Other manic episodes |
|  | F309 | Manic episode, unspecified |
|  | F310 | Bipolar disorder, current episode hypomanic |
|  | F3110 | Bipolar disorder, current episode manic without psychotic features, unspecified |
|  | F3111 | Bipolar disorder, current episode manic without psychotic features, mild |
|  | F3112 | Bipolar disorder, current episode manic without psychotic features, moderate |
|  | F3113 | Bipolar disorder, current episode manic without psychotic features, severe |
|  | F312 | Bipolar disorder, current episode manic severe with psychotic features |
|  | F3130 | Bipolar disorder, current episode depressed, mild or moderate severity, unspecified |
|  | F3131 | Bipolar disorder, current episode depressed, mild |
|  | F3132 | Bipolar disorder, current episode depressed, moderate |
|  | F314 | Bipolar disorder, current episode depressed, severe, without psychotic features |
|  | F315 | Bipolar disorder, current episode depressed, severe, with psychotic features |
|  | F3160 | Bipolar disorder, current episode mixed, unspecified |
|  | F3161 | Bipolar disorder, current episode mixed, mild |
|  | F3162 | Bipolar disorder, current episode mixed, moderate |
|  | F3163 | Bipolar disorder, current episode mixed, severe, without psychotic features |
|  | F3164 | Bipolar disorder, current episode mixed, severe, with psychotic features |
|  | F3170 | Bipolar disorder, currently in remission, most recent episode unspecified |
|  | F3171 | Bipolar disorder, in partial remission, most recent episode hypomanic |
|  | F3172 | Bipolar disorder, in full remission, most recent episode hypomanic |
|  | F3173 | Bipolar disorder, in partial remission, most recent episode manic |
|  | F3174 | Bipolar disorder, in full remission, most recent episode manic |
|  | F3175 | Bipolar disorder, in partial remission, most recent episode depressed |
|  | F3176 | Bipolar disorder, in full remission, most recent episode depressed |
|  | F3177 | Bipolar disorder, in partial remission, most recent episode mixed |
|  | F3178 | Bipolar disorder, in full remission, most recent episode mixed |
|  | F3181 | Bipolar II disorder |
|  | F3189 | Other bipolar disorder |
|  | F319 | Bipolar disorder, unspecified |
|  | F320 | Major depressive disorder, single episode, mild |
|  | F321 | Major depressive disorder, single episode, moderate |
|  | F322 | Major depressive disorder, single episode, severe without psychotic features |
|  | F323 | Major depressive disorder, single episode, severe with psychotic features |
|  | F324 | Major depressive disorder, single episode, in partial remission |
|  | F325 | Major depressive disorder, single episode, in full remission |
|  | F3281 | Premenstrual dysphoric disorder |
|  | F3289 | Single episode of 'masked' depression, not otherwise specified |
|  | F329 | Major depressive disorder, single episode, unspecified |
|  | F330 | Major depressive disorder, recurrent, mild |


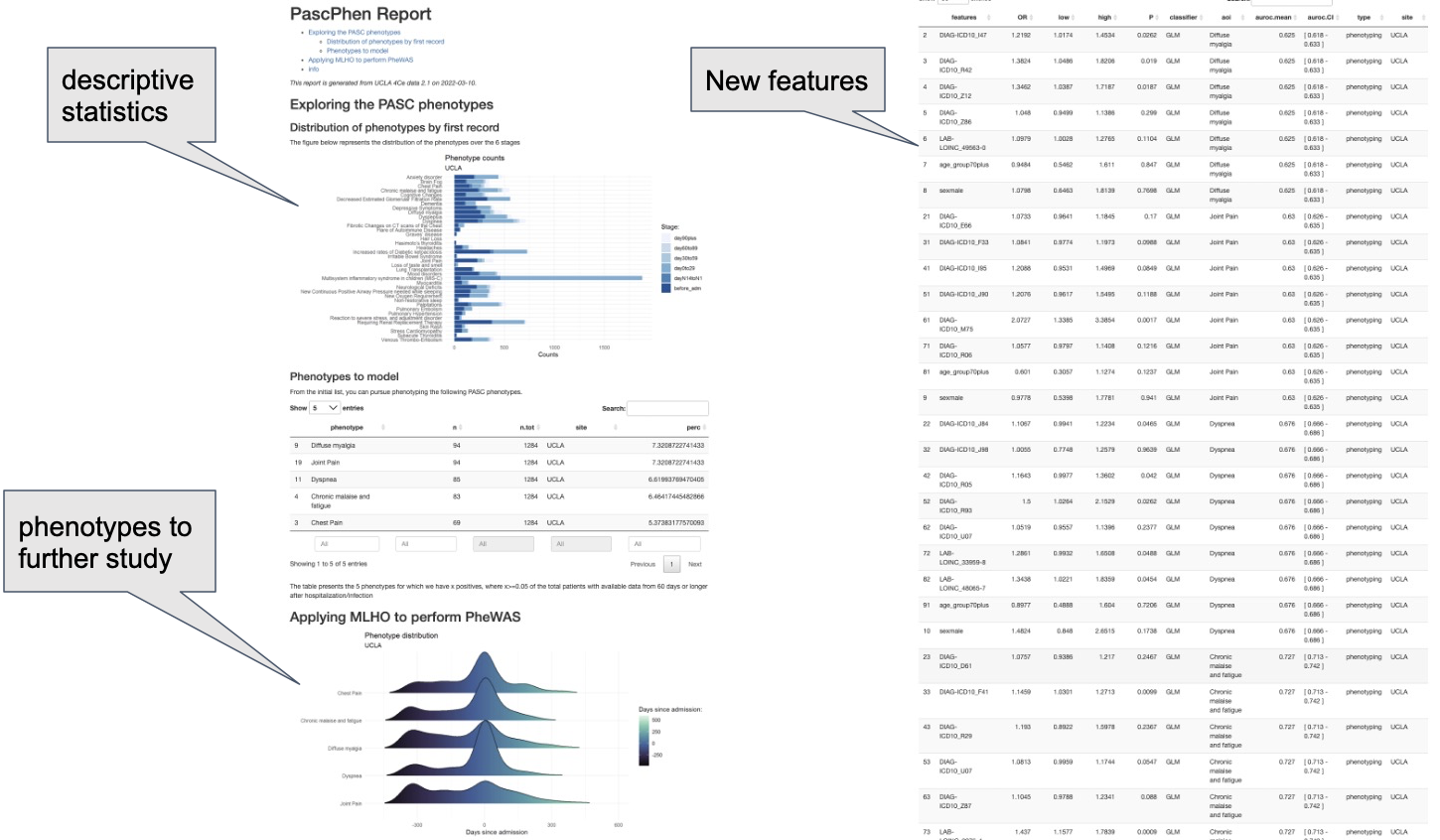


**Figure S2. Harmonized HTML output resulting from phase 1 implementation of the PascPhen package across 4CE sites.** The report consisted of 3 panels providing descriptive statistics about the initial list of subtypes, temporal distribution of subtypes with a minimum prevalence in the patient population, and new features identified by iterative MLHO implementations that associated with the core features.

**Table S2. Complete list of augmented features identified by MLHO that are suggestive of dyspnea, chronic fatigue, and joint pain.**

|  | | **PASC Subtype** | | |
| --- | --- | --- | --- | --- |
|  | | **Dyspnea** | **Chronic fatigue** | **Joint pain** |
| Core Features | | R06.* - Abnormalities in Breathing | F53.* - Fatigue | M25.5* Pain in Joint |
| Sample  Augmented Features | Diseases with related symptom | I48.0 - Paroxysmal Atrial Fibrillation  I48.91 - Atrial fibrillation, unspecified  I50.9 - Heart failure, unspecified  I51.7 - Cardiology  G62.9 - Polyneuropathy, unspecified  G72.81 - Critical Illness myopathy  I25.10 - Atherosclerotic heart disease of native coronary artery | E03.9 - Hypothyroidism, Unspecified  F32.A - Depression, Unspecified  G47.33 Obstructive sleep apnea  G72.81 - Critical Illness myopathy | M17.0 - Bilateral primary osteoarthritis of the knee  E03.9 Hypothyroidism, unspecified  E55.9 - Vitamin D deficiency, unspecified  M17.11 Unilateral primary osteoarthritis, right knee  M17.12 Unilateral primary osteoarthritis, left knee |
|  | Laboratory tests suggestive of symptom | LNC 48065-7 D-dimer  LNC 6598-7 Troponin T  LNC 49563 Troponin I  LNC 2276-4 Ferritin | LNC 2276-4 Ferritin  LNC 49563-0 Troponin I  LNC 6598-7 Troponin T | LNC 1988-5 CRP |
|  | Procedures suggestive of symptom | CT Chest | - | - |
|  | Synonyms | R09.02 - Hypoxemia | - | M54.5 - Low Back Pain |

*Includes all ICD codes within the parental group

**Table S3. Complete list of augmented features identified by MLHO that are suggestive of anxiety, dyspepsia, neurological deficits, and chest pain**

|  | | **PASC Subtype** | | | |
| --- | --- | --- | --- | --- | --- |
|  | | **Anxiety** | **Dyspepsia** | **Neurological deficits** | **Chest pain** |
| Core Features | | R41.* Anxiety Disorders | K30 - Functional dyspepsia  K21.0 - GERD with esophagitis  K21.9 - GERD without esophagitis | R25.* - Abnormal involuntary movements  R26.* - Abnormalities of gait and mobility  R27.* - Other lack of coordination  R29.* - Other symptoms and signs involving the nervous and musculoskeletal symptems | R07.1 - Chest pain on breathing  R07.2 - Precordial pain  R07.81 - Pleurodynia  R07.82 - Intercostal pain  R07.89 - Other chest pain  R07.9 Chest pain, unspecified |
| Sample  Augmented Features | Diseases with related symptom | F32.9 - Major depressive disorder, single episode, unspecified  F32.A - Depression, unspecified  R07.89 - Other chest pain | - | - | - |
|  | Laboratory tests suggestive of symptom | - | - | - | LNC 48065-7 D-dimer  LNC 49563 - Troponin |
|  | Procedures suggestive of symptom | - | - | - | - |
|  | Synonyms | F43.10 - Post traumatic stress disorder, unspecified | R10.13 Epigastric pain | R53.1 Weakness  R41.0 Disorientation, unspecified  R41.82 - Altered mental status, unspecified | I20.8 Other forms of angina pectoris  I49.9 - Cardiac arrhythmia, unspecified  R00.2 - Palpitations |

*Includes all ICD codes within the parental group


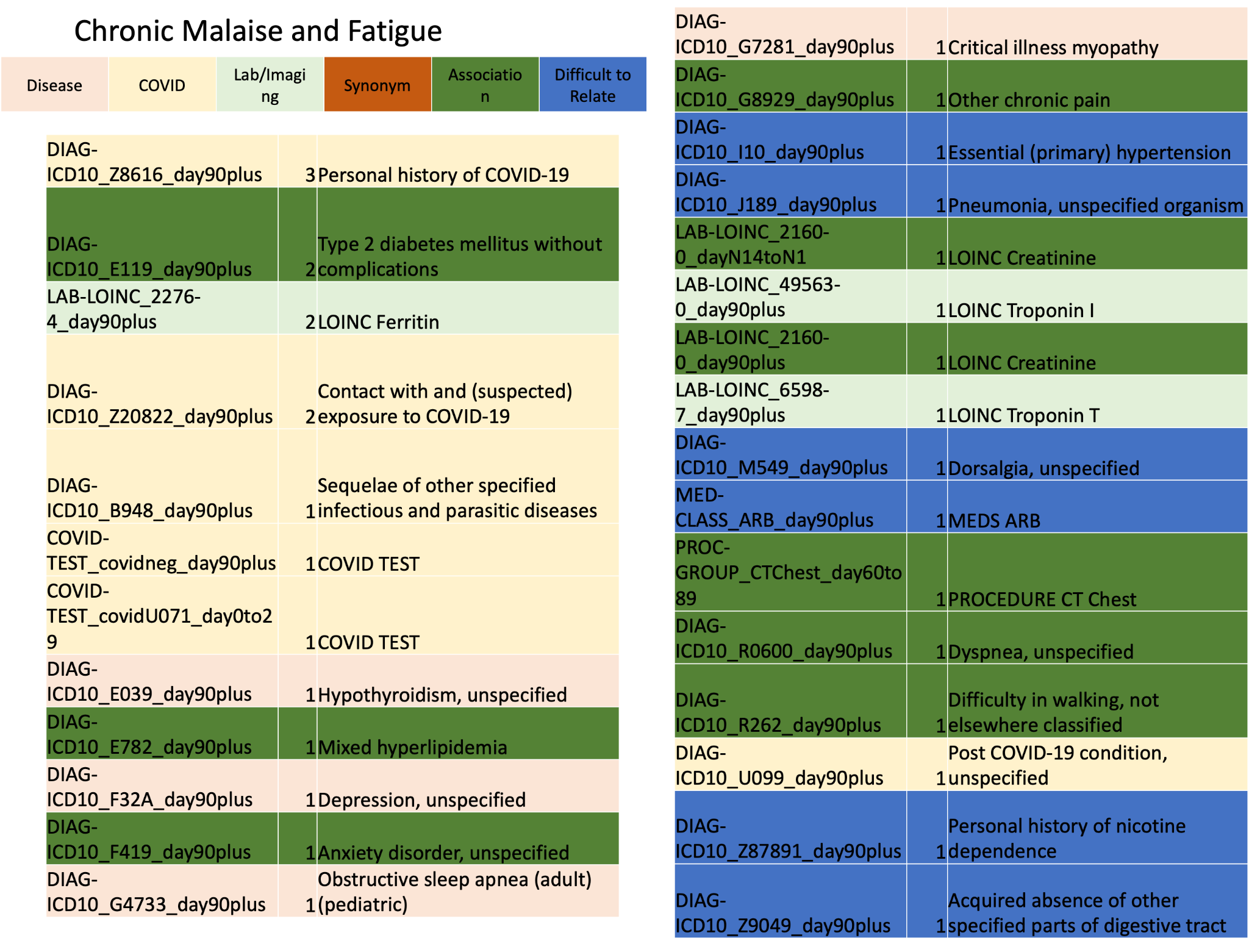

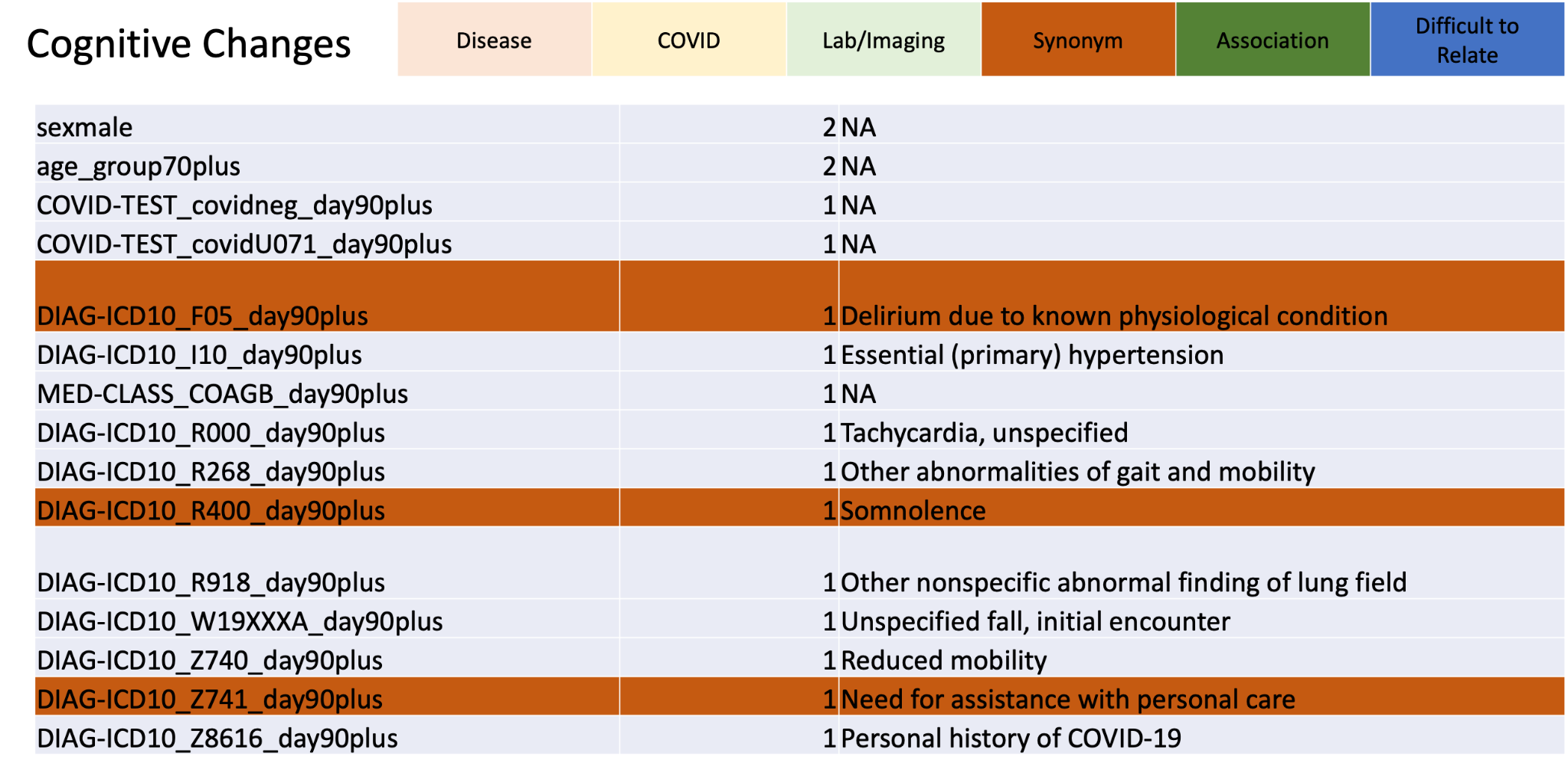

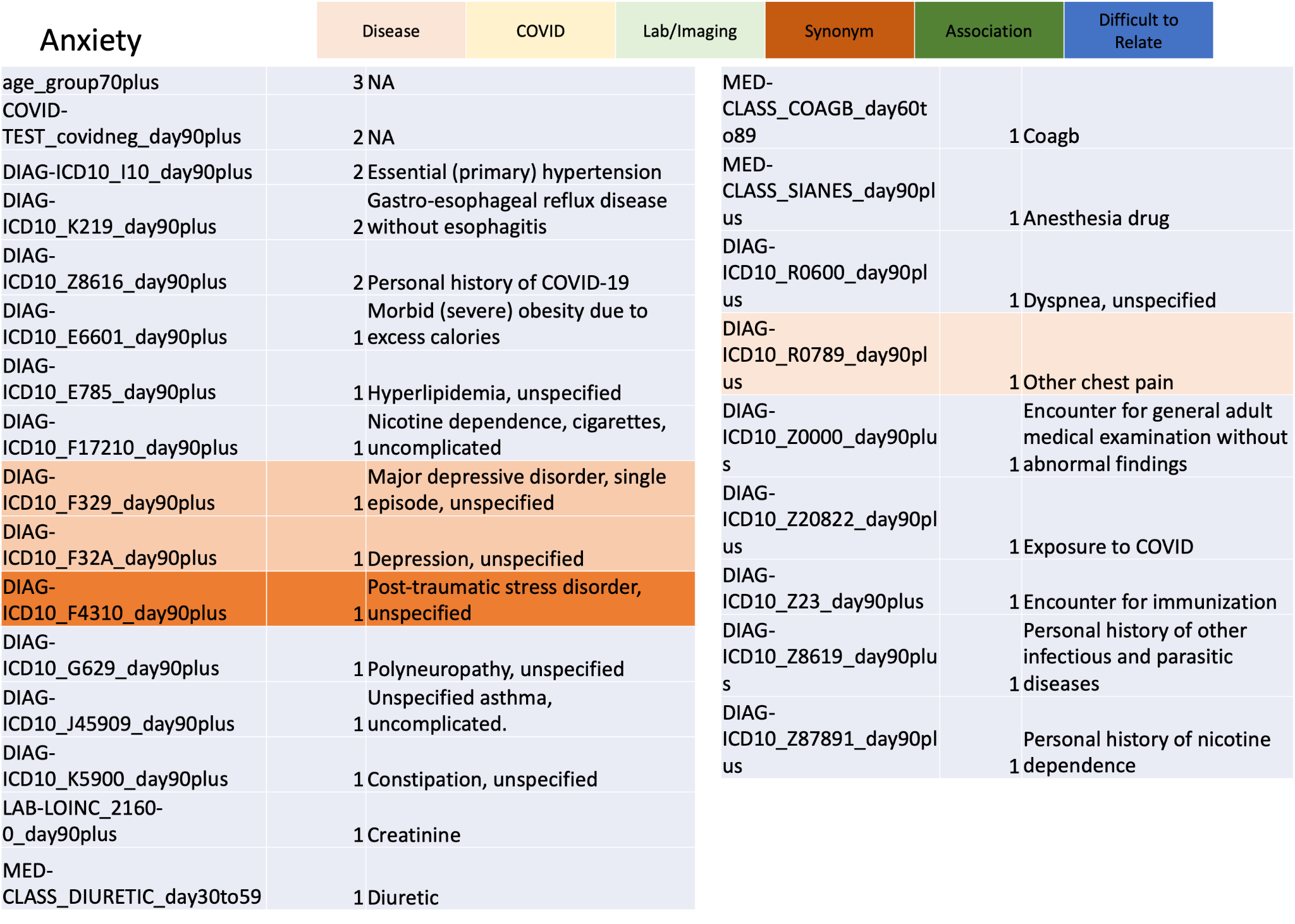


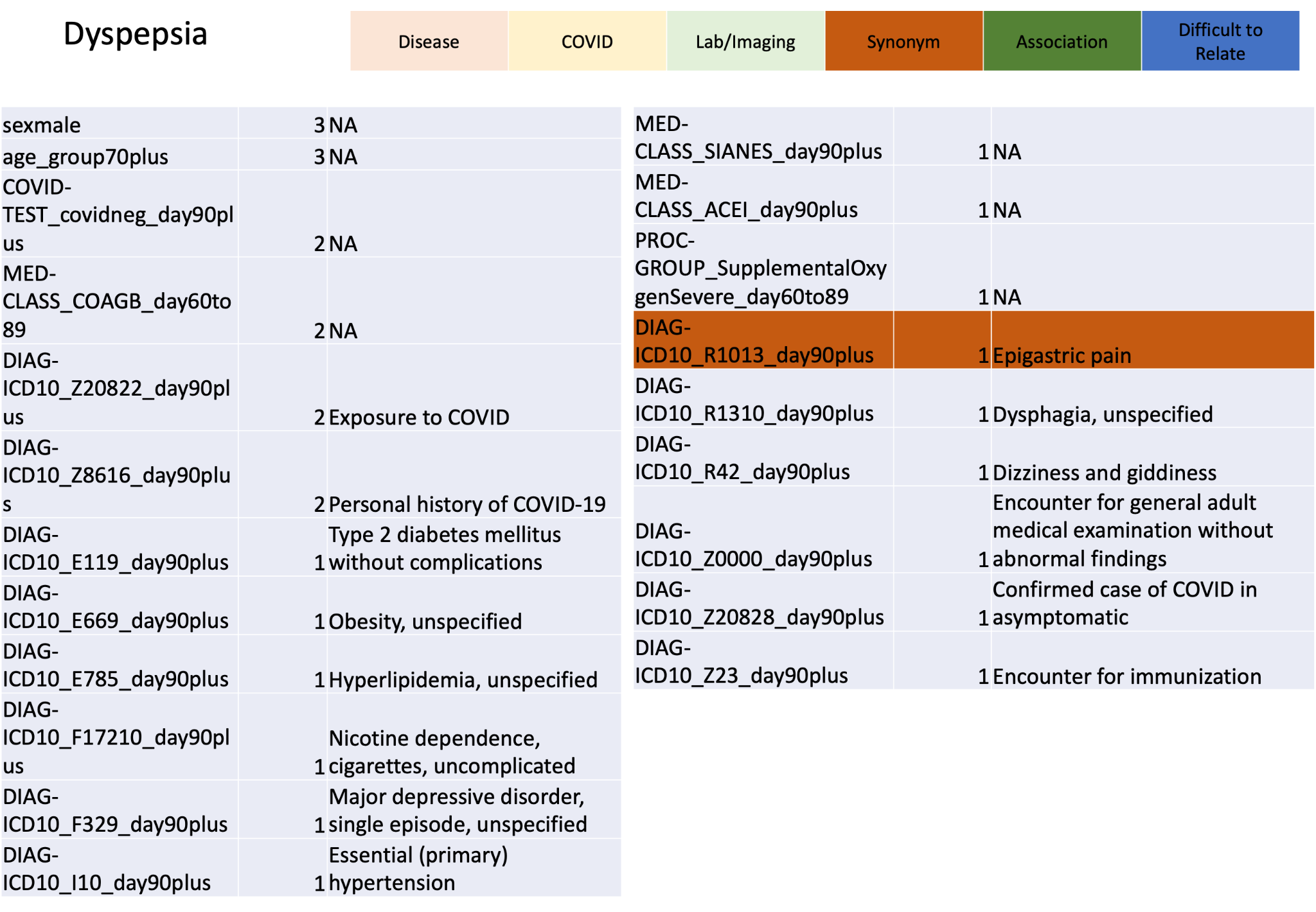


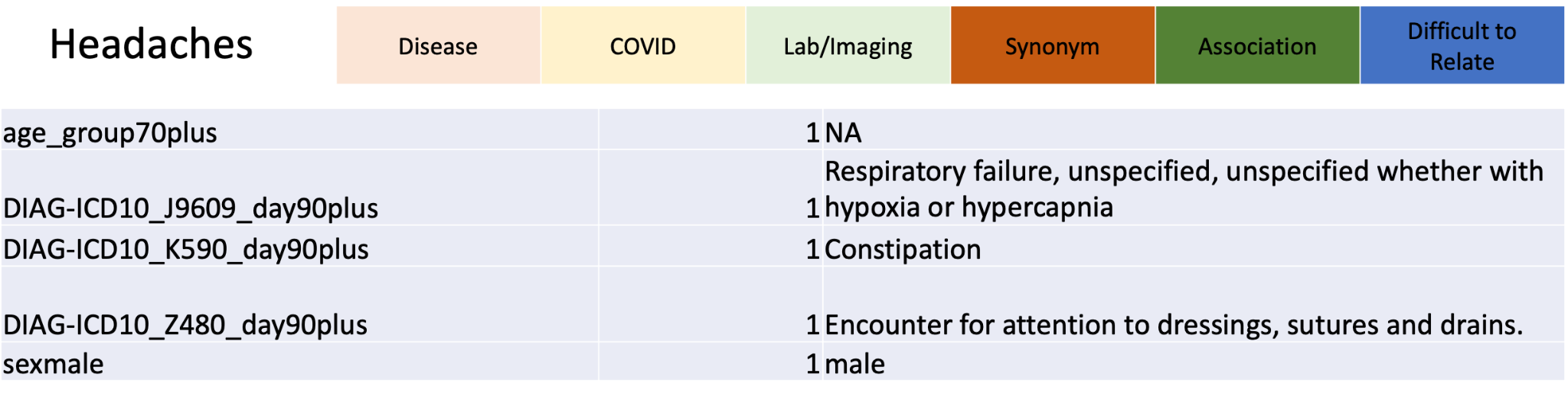


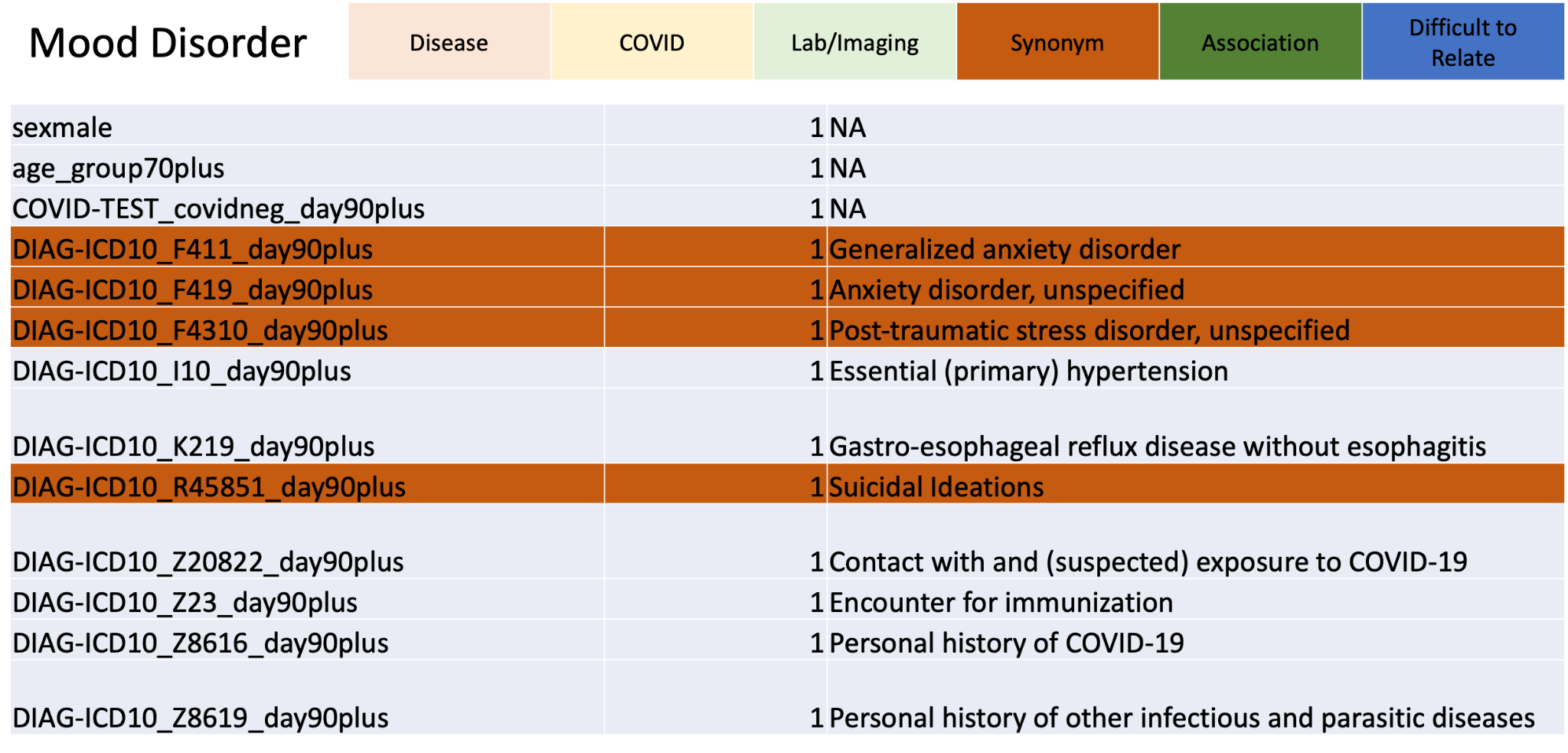


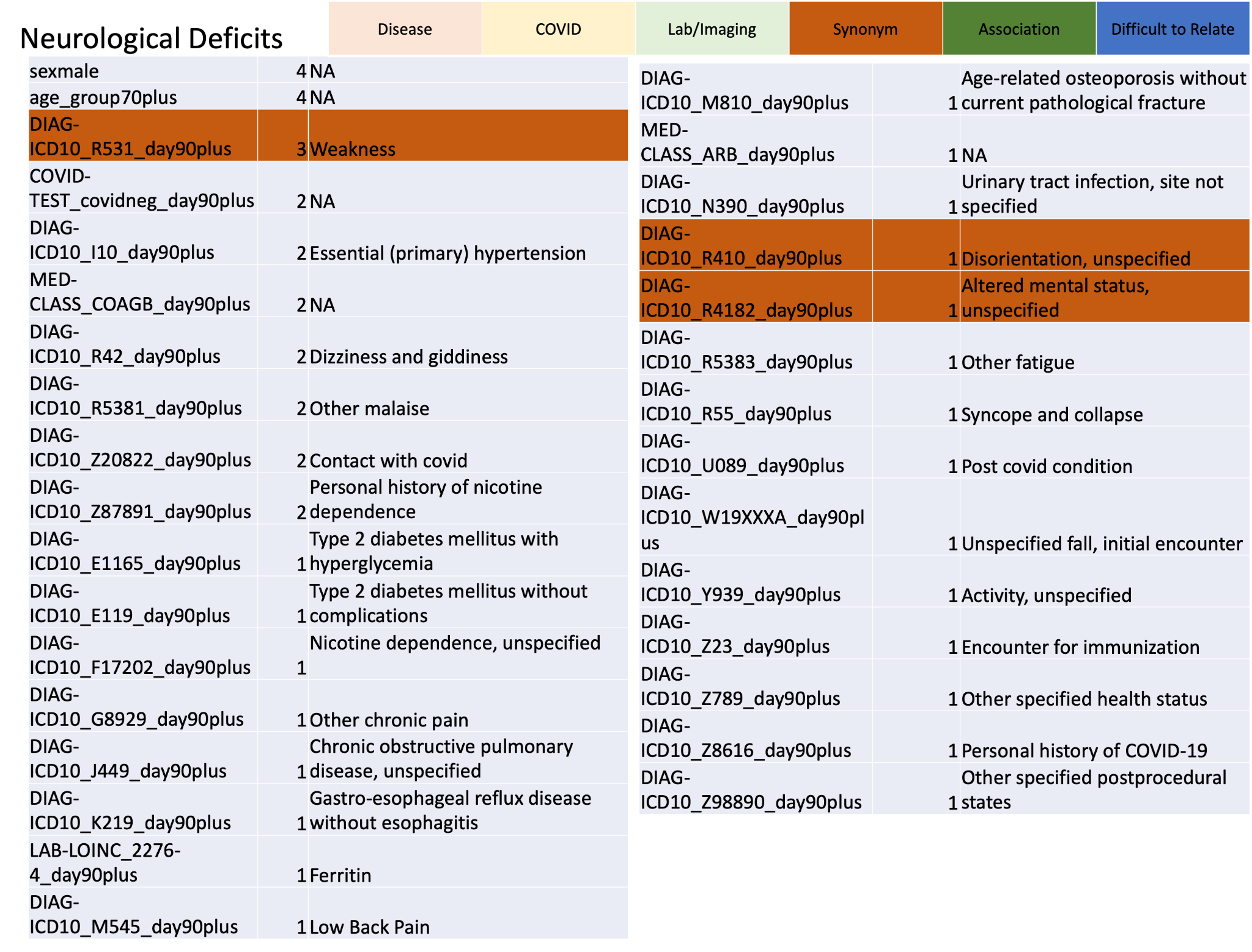


**Figure S3. Clinical classification of new features identified by MLHO for 7 after COVID-19 infection.** The new features are identified by MLHO across multiple hospital systems to associate with the core features for chest pain at different time points.


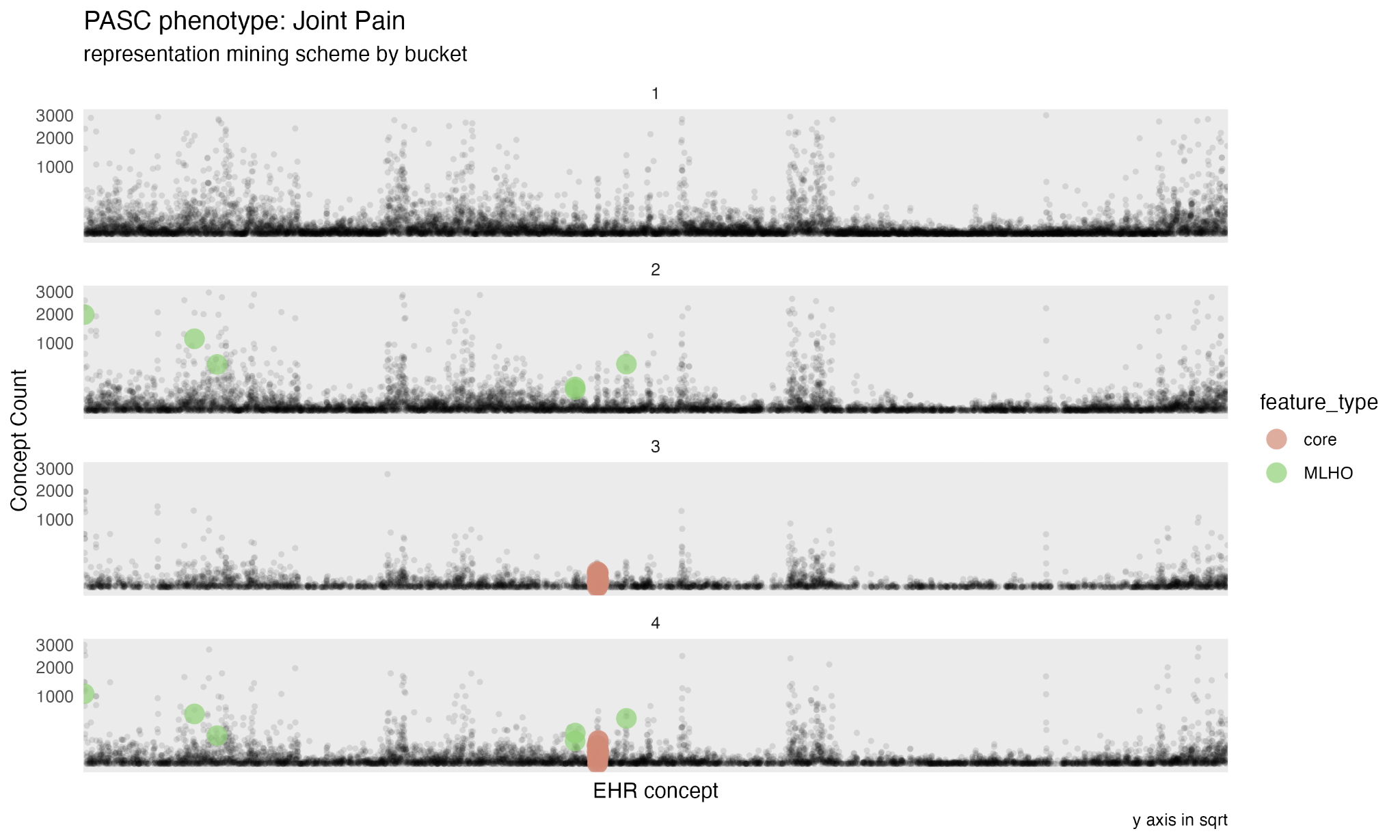


**Figure S4. Signal detection schema for long COVID joint pain.** Figure shows the incremental progress from identifying signals among noise to identify patients with long COVID complications.

**Table S4. Prevalence estimates for PASC subtypes - weighted averages via number of patients enrolled by each hospital system.**

| **PASC Subtype** | **Average (%)** | **Upper Limit (%)** | **Lower Limit (%)** |
| --- | --- | --- | --- |
| **One or more** | 15.58 | 20.03 | 11.12 |
| **More than one** | 5.98 | 7.90 | 4.06 |
| **Joint pain** | 5.45 | 6.76 | 4.14 |
| **Dyspnea** | 4.53 | 5.09 | 3.96 |
| **Chronic malaise and fatigue** | 4.41 | 5.30 | 3.51 |
| **Chest pain** | 3.42 | 4.19 | 2.65 |
| **Neurological deficits** | 3.28 | 4.96 | 1.60 |
| **Dyspepsia** | 1.38 | 2.50 | 0.25 |
| **Cognitive changes** | 1.72 | 2.97 | 0.47 |

**Table S5. Prevalence estimates for PASC subtypes - upper and lower limits for each hospital system.**

| **site** | **PASC Subtype** | **Upper Limit (count)** | **Upper Limit (perc)** | **Lower Limit (count)** | **Lower Limit (perc)** |
| --- | --- | --- | --- | --- | --- |
| **Hosp 1** | *At Least 1* | *290* | *16.00* | *133* | *7.00* |
| **Hosp 1** | Chest Pain | 45 | 2.53 | 4 | 0.23 |
| **Hosp 1** | Chronic malaise and fatigue | 85 | 5.19 | 51 | 3.27 |
| **Hosp 1** | Cognitive Changes | 29 | 2.03 | 1 | 0.06 |
| **Hosp 1** | Dyspepsia | 58 | 3.27 | 3 | 0.17 |
| **Hosp 1** | Dyspnea | 84 | 5.13 | 61 | 3.43 |
| **Hosp 1** | Joint Pain | 78 | 4.39 | 38 | 2.14 |
| **Hosp 1** | *Multiple PASC* | *96* | *5.00* | *30* | *2.00* |
| **Hosp 1** | Neurological Deficits | 43 | 2.42 | 11 | 1.02 |
| **Hosp 2** | *At Least 1* | *2266* | *28.00.00* | *1415* | *17.00* |
| **Hosp 2** | Chest Pain | 612 | 7.48 | 401 | 5.30 |
| **Hosp 2** | Chronic malaise and fatigue | 657 | 8.03 | 398 | 5.27 |
| **Hosp 2** | Cognitive Changes | 420 | 5.14 | 112 | 1.37 |
| **Hosp 2** | Dyspepsia | 248 | 3.03 | 49 | 1.00 |
| **Hosp 2** | Dyspnea | 625 | 8.04 | 484 | 6.32 |
| **Hosp 2** | Joint Pain | 899 | 11.39 | 570 | 7.37 |
| **Hosp 2** | *Multiple PASC* | *1038* | *13.00* | *541* | *7.00* |
| **Hosp 2** | Neurological Deficits | 529 | 6.47 | 229 | 3.20 |
| **Hosp 3** | *At Least 1* | *3198* | *17.00* | *1767* | *9.00* |
| **Hosp 3** | Chest Pain | 564 | 3.34 | 366 | 2.31 |
| **Hosp 3** | Chronic malaise and fatigue | 803 | 4.19 | 574 | 3.39 |
| **Hosp 3** | Cognitive Changes | 416 | 2.17 | 25 | 0.13 |
| **Hosp 3** | Dyspepsia | 423 | 2.21 | 21 | 0.11 |
| **Hosp 3** | Dyspnea | 774 | 4.04 | 608 | 3.17 |
| **Hosp 3** | Joint Pain | 993 | 5.18 | 598 | 3.12 |
| **Hosp 3** | *Multiple PASC* | *1142* | *6.00* | *480* | *3.00* |
| **Hosp 3** | Neurological Deficits | 872 | 4.55 | 226 | 1.18 |

**S1. PASC Temporal Distributions comparison**

**S1.1. Overall Results**

We compare PASC temporal distributions by means of Kruskal-Wallis rank sum test

and Wilcoxon rank sum test with Bonferroni correction.

**Kruskal-Wallis rank sum test**

Kruskal-Wallis chi-squared = 73.227, df = 6, p-value = 8.89e-14

**Pairwise comparisons using Wilcoxon rank sum test with continuity correction**

Pairwise comparisons using Wilcoxon rank sum test with continuity correction

P value adjustment method: Bonferroni

**Table S6.** Wilcoxon rank sum test overall results.

|  | **Chest pain** | **Chronic malaise and fatigue** | **Cognitive changes** | **Dyspepsia** | **Dyspnea** | **Joint pain** |
| --- | --- | --- | --- | --- | --- | --- |
| **Chronic malaise and fatigue** | 1 |  |  |  |  |  |
| **Cognitive changes** | ***0.0012*** | ***0.00019*** |  |  |  |  |
| **Dyspepsia** | 0.43 | 0.21 | 1 |  |  |  |
| **Dyspnea** | 1 | 1 | ***2.68e-05*** | 0.13 |  |  |
| **Joint pain** | 1 | 1 | ***0.019*** | 1 | 0.574 |  |
| **Neurological deficits** | ***1.55e-06*** | ***3.78e-08*** | 1 | 1 | ***2.18e-10*** | ***2.18e-05*** |

**S1.2. Stratified analysis**

We consider site effects when comparing PASC subtype distributions in time, hereafter the Wilcoxon rank sum test results for each site.

**Table S7.** Wilcoxon rank sum test hospital system 1 results.

|  | **Chest pain** | **Chronic malaise and fatigue** | **Cognitive changes** | **Dyspepsia** | **Dyspnea** | **Joint pain** |
| --- | --- | --- | --- | --- | --- | --- |
| **Chronic malaise and fatigue** | 1 |  |  |  |  |  |
| **Cognitive changes** | 1 | 0.33 |  |  |  |  |
| **Dyspepsia** | 1 | 1 | 1 |  |  |  |
| **Dyspnea** | 1 | 1 | 0.26 | 1 |  |  |
| **Joint Pain** | 1 | 1 | 0.28 | 1 | 1 |  |
| **Neurological deficits** | 1 | 0.39 | 1 | 1 | 0.337 | 0.40 |

**Table S8.** Wilcoxon rank sum test hospital system 2 results.

|  | **Chest pain** | **Chronic malaise and fatigue** | **Cognitive changes** | **Dyspepsia** | **Dyspnea** | **Joint pain** |
| --- | --- | --- | --- | --- | --- | --- |
| **Chronic malaise and fatigue** | 1 |  |  |  |  |  |
| **Cognitive Changes** | 0.12 | 0.34 |  |  |  |  |
| **Dyspepsia** | 1 | 1 | 1 |  |  |  |
| **Dyspnea** | 1 | 1 | 0.17 | 1 |  |  |
| **Joint pain** | 0.85 | 1 | 1 | 1 | 1 |  |
| **Neurological deficits** | ***0.0025*** | ***0.014*** | 1 | 1 | ***0.0024*** | 0.52 |

**Table S9.** Wilcoxon rank sum test hospital system 3 results.

|  | **Chest pain** | **Chronic malaise and fatigue** | **Cognitive changes** | **Dyspepsia** | **Dyspnea** | **Joint pain** |
| --- | --- | --- | --- | --- | --- | --- |
| **Chronic malaise and fatigue** | 0.06 |  |  |  |  |  |
| **Cognitive changes** | 0.20 | ***1.81e-06*** |  |  |  |  |
| **Dyspepsia** | 1 | 0.06 | 1 |  |  |  |
| **Dyspnea** | ***0.02*** | 1 | ***1.61e-07*** | ***0.041*** |  |  |
| **Joint pain** | 1 | 0.22 | ***0.003*** | 1 | 0.064 |  |
| **Neurological deficits** | 0.06 | ***1.54e-09*** | 1 | 1 | ***5.11e-12*** | ***6.86e-05*** |

**Table S10. Features temporal distribution for PASC subtype features.**

| **PASC Subtype** | **Total Features Count** | **Mean Time** | **SD Time** | **Median Time** | **Q25 Time** | **Q75 Time** |
| --- | --- | --- | --- | --- | --- | --- |
| **Chest pain** | 11,508 | 327 | 156 | 310 | 200 | 440 |
| **Chronic malaise and fatigue** | 22,572 | 330 | 158 | 310 | 200 | 450 |
| **Dyspnea** | 41,174 | 330 | 157 | 310 | 200 | 450 |
| **Joint pain** | 20,807 | 319 | 154 | 300 | 190 | 430 |
| **Neurological deficits** | 7,300 | 289 | 139 | 270 | 170 | 390 |
| **Cognitive changes** | 2,018 | 294 | 144 | 270 | 170 | 400 |
| **Dyspepsia** | 1,669 | 303 | 147 | 280 | 180 | 410 |

**Table S11. Features temporal distribution for PASC subtype features in each site**

| **PASC Subtype** | **site** | **Count** | **AvgTime** | **SDTime** | **Median** | **Q25** | **Q75** |
| --- | --- | --- | --- | --- | --- | --- | --- |
| Chest Pain | Hosp 2 | 5204 | 350 | 162 | 340 | 210 | 480 |
| Chest Pain | Hosp 1 | 85 | 267 | 145 | 250 | 160 | 330 |
| Chest Pain | Hosp 3 | 6219 | 307 | 143 | 295 | 190 | 410 |
| Chronic malaise and fatigue | Hosp 2 | 6944 | 347 | 162 | 330 | 210 | 480 |
| Chronic malaise and fatigue | Hosp 1 | 761 | 282 | 149 | 250 | 160 | 380 |
| Chronic malaise and fatigue | Hosp 3 | 14867 | 339 | 155 | 330 | 210 | 460 |
| Cognitive Changes | Hosp 2 | 1296 | 317 | 150 | 300 | 190 | 435 |
| Cognitive Changes | Hosp 1 | 38 | 215 | 107 | 200 | 140 | 250 |
| Cognitive Changes | Hosp 3 | 684 | 277 | 135 | 260 | 162 | 370 |
| Dyspepsia | Hosp 2 | 940 | 328 | 155 | 310 | 200 | 460 |
| Dyspepsia | Hosp 1 | 75 | 248 | 123 | 230 | 160 | 310 |
| Dyspepsia | Hosp 3 | 654 | 293 | 141 | 280 | 170 | 395 |
| Dyspnea | Hosp 2 | 16011 | 346 | 160 | 340 | 210 | 480 |
| Dyspnea | Hosp 1 | 1173 | 277 | 140 | 250 | 170 | 360 |
| Dyspnea | Hosp 3 | 23990 | 340 | 155 | 330 | 210 | 460 |
| Joint Pain | Hosp 2 | 8633 | 332 | 160 | 310 | 200 | 460 |
| Joint Pain | Hosp 1 | 810 | 275 | 137 | 250 | 170 | 350 |
| Joint Pain | Hosp 3 | 11364 | 317 | 149 | 300 | 190 | 422 |
| Neurological Deficits | Hosp 2 | 4636 | 311 | 150 | 290 | 190 | 420 |
| Neurological Deficits | Hosp 1 | 119 | 238 | 125 | 210 | 140 | 307 |
| Neurological Deficits | Hosp 3 | 2545 | 276 | 127 | 260 | 170 | 370 |


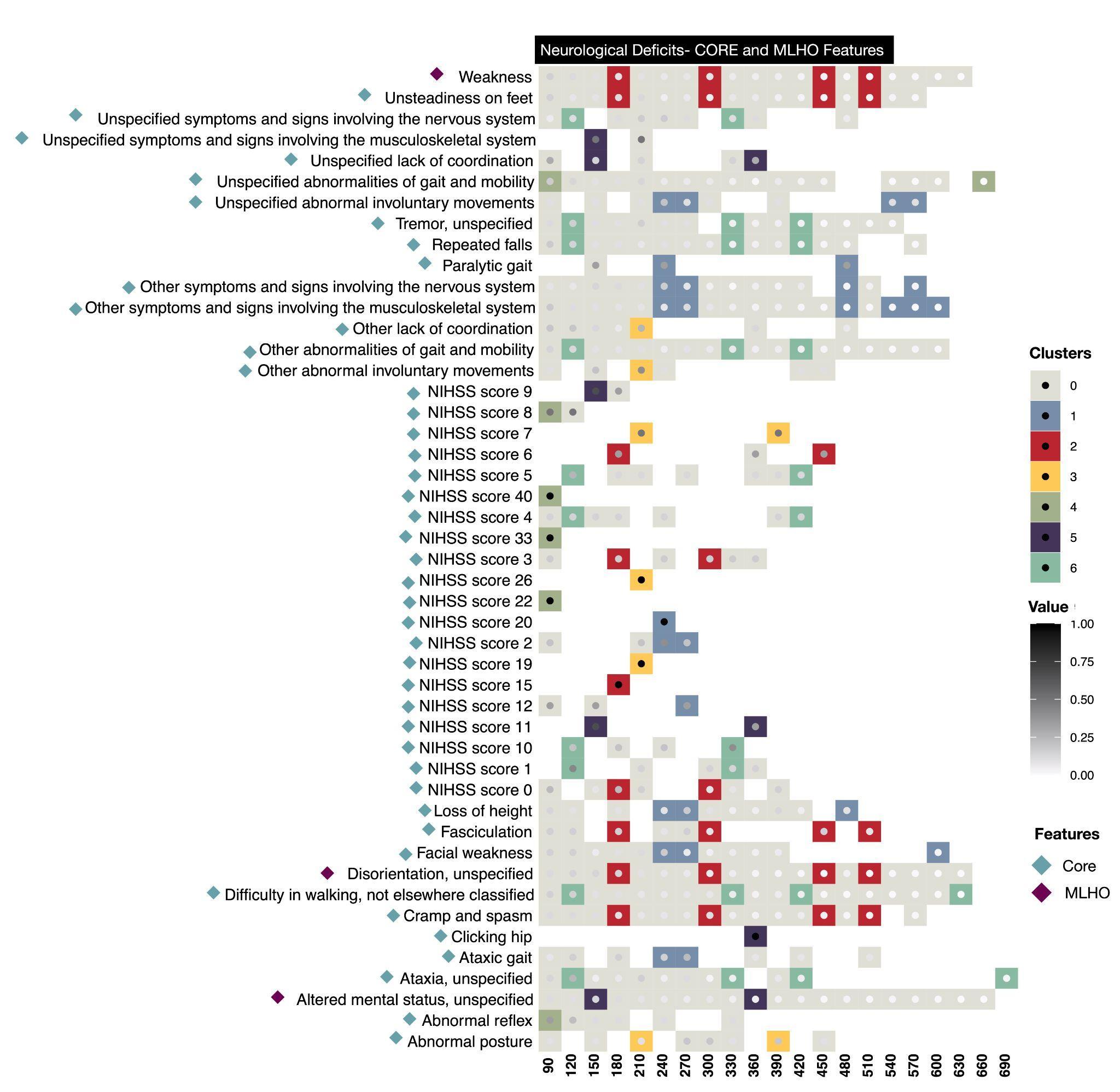


**Figure S5**. **Neurological deficits–Clustered presentation and temporal distribution of the core and MLHO features.** Sparklines are available [here](https://drive.google.com/uc?id=1tQ35Wi7ZvMghLYPZP2TgUroYHfs-KMK-&export=download).


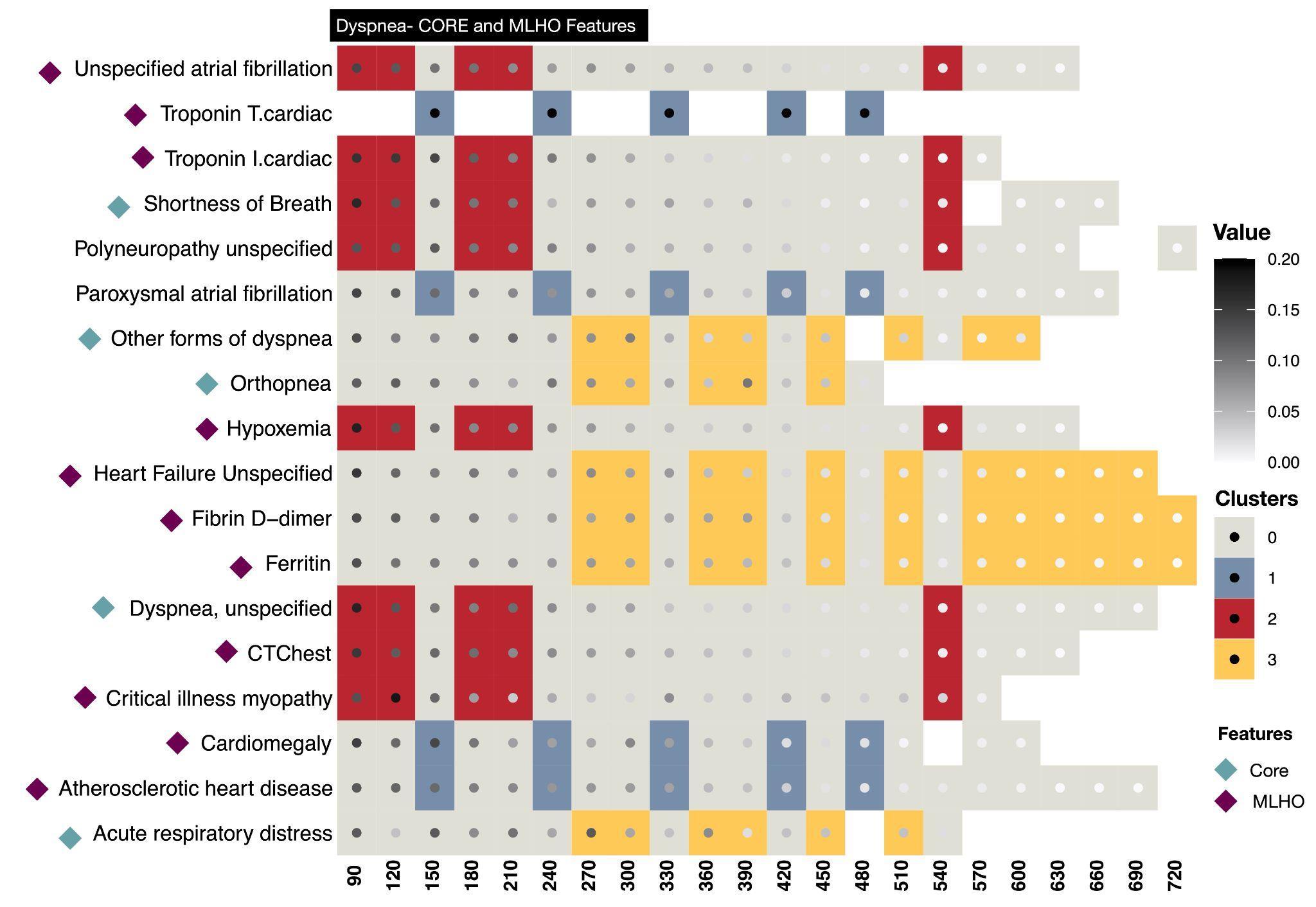


**Figure S6**. **Dyspnea– Clustered presentation and temporal distribution of the core and MLHO features.** Sparklines are available [here](https://drive.google.com/uc?id=1Jits9pcbjOvxiha5APL1tt-ej-y_Bqbp&export=download).


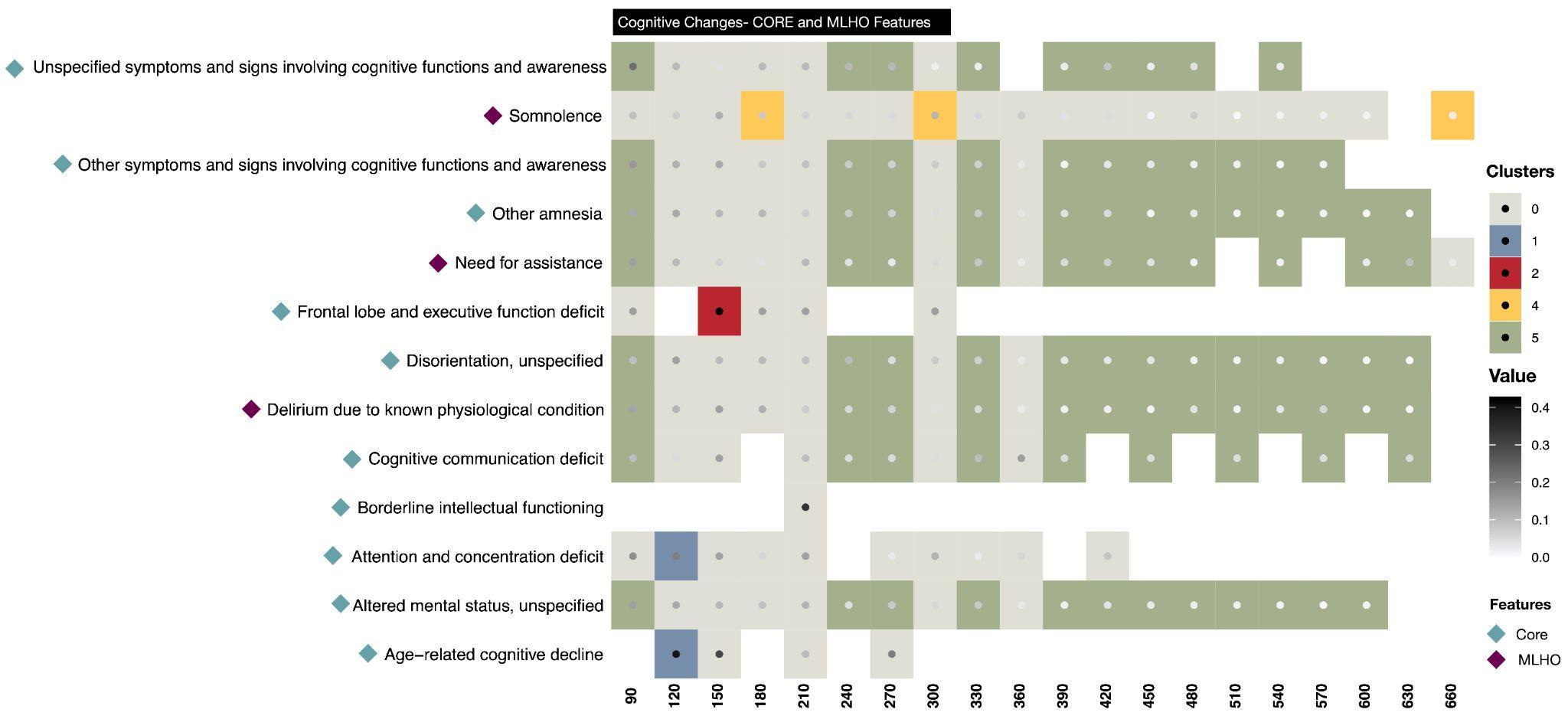


**Figure S7**. **Cognitive changes–Clustered presentation and temporal distribution of the core and MLHO features.** Sparklines are available [here](https://drive.google.com/uc?id=1G6okm2m73wN-7MkzkXgLRqSNOhUreKrt&export=download).


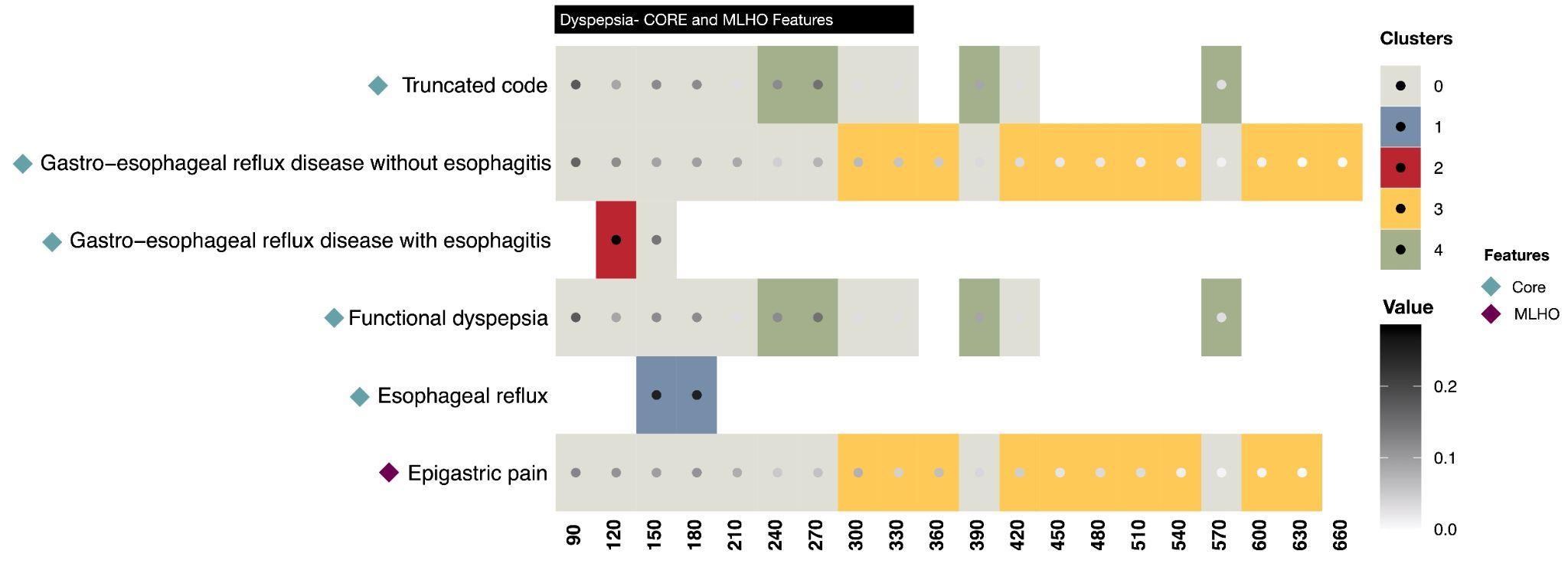


**Figure S8**. **Dyspepsia – Clustered presentation and temporal distribution of the core and MLHO features.** Sparklines are available [here](https://drive.google.com/uc?id=1LFVX8LjgD2k60HxN-inwvmeY2e-kg2e1&export=download).
