# Supplementary material for: Characterization of long COVID temporal sub-phenotypes by distributed representation learning from electronic health record data: a cohort study
Source: eClinicalMedicine. 2023 Sep 14;64:102210. doi: 10.1016/j.eclinm.2023.102210 (PMC10511779; doi:10.1016/j.eclinm.2023.102210)
Supplement: 4ceMembers [file mmc2.docx]

Supplementary Note 1: Consortium for Clinical Characterization of COVID-19 by EHR (4CE) Members and Affiliations

James R Aaron40, Giuseppe Agapito41, Adem Albayrak42, Giuseppe Albi29, Mario Alessiani43, Anna Alloni38, Danilo F Amendola44, François Angoulvant45, Li L.L.J Anthony46, Bruce J Aronow33, Fatima Ashraf47, Andrew Atz48, Paul Avillach1, Paula S Azevedo49, James Balshi50, Brett K Beaulieu-Jones1, Douglas S Bell24, Antonio Bellasi51, Riccardo Bellazzi29, Vincent Benoit25, Michele Beraghi52, José Luis Bernal-Sobrino53, Mélodie Bernaux54, Romain Bey25, Surbhi Bhatnagar33, Alvar Blanco-Martínez53, Clara-Lea Bonzel1, John Booth55, Silvano Bosari36, Florence T Bourgeois12, Robert L Bradford56, Gabriel A Brat1, Stéphane Bréant57, Nicholas W Brown1, Raffaele Bruno58, William A Bryant55, Mauro Bucalo38, Emily Bucholz59, Anita Burgun60, Tianxi Cai1, Mario Cannataro61, Aldo Carmona62, Charlotte Caucheteux63, Julien Champ64, Jin Chen65, Krista Y Chen66, Luca Chiovato32, Lorenzo Chiudinelli67, Kelly Cho30, James J Cimino68, Tiago K Colicchio68, Sylvie Cormont57, Sébastien Cossin31, Jean B Craig69, Juan Luis Cruz-Bermúdez53, Jaime Cruz-Rojo53, Arianna Dagliati2, Mohamad Daniar70, Christel Daniel71, Priyam Das1, Batsal Devkota72, Audrey Dionne59, Rui Duan3, Julien Dubiel57, Scott L DuVall73, Loic Esteve74, Hossein Estiri16, Shirley Fan75, Robert W Follett24, Thomas Ganslandt76, Noelia García- Barrio53, Lana X Garmire77, Nils Gehlenborg1, Emily J Getzen78, Alon Geva79, Tobias Gradinger76, Alexandre Gramfort63, Romain Griffier31, Nicolas Griffon71, Olivier Grisel63, Alba Gutiérrez-Sacristán1, Larry Han3, David A Hanauer9, Christian Haverkamp80, Derek Y Hazard81, Bing He77, Darren W Henderson40, Martin Hilka57, Yuk-Lam Ho23, John H Holmes10,11, Chuan Hong6,1, Kenneth M Huling1, Meghan R Hutch82, Richard W Issitt55, Anne Sophie Jannot83, Vianney Jouhet31, Ramakanth Kavuluru26, Mark S Keller1, Chris J Kennedy84, Daniel A Key55, Katie Kirchoff85, Jeffrey G Klann16, Isaac S Kohane1, Ian D Krantz86, Detlef Kraska87, Ashok K Krishnamurthy88, Sehi L'Yi1, Trang T Le10, Judith Leblanc89, Guillaume Lemaitre63, Leslie Lenert69, Damien Leprovost90, Molei Liu91, Ne Hooi Will Loh92, Qi Long93, Sara Lozano-Zahonero21, Yuan Luo82, Kristine E Lynch73, Sadiqa Mahmood42, Sarah E Maidlow13, Adeline Makoudjou21, Alberto Malovini28, Kenneth D Mandl66, Chengsheng Mao82, Anupama Maram94, Patricia Martel95, Marcelo R Martins96, Jayson S Marwaha97, Aaron J Masino98, Maria Mazzitelli99, Arthur Mensch100, Marianna Milano101, Marcos F Minicucci102, Bertrand Moal14, Taha Mohseni Ahooyi103, Jason H Moore104, Cinta Moraleda105, Jeffrey S Morris106, Michele Morris7, Karyn L Moshal107, Sajad Mousavi1, Danielle L Mowery10, Douglas A Murad24, Shawn N Murphy15, Thomas P Naughton108, Carlos Tadeu Breda Neto44, Antoine Neuraz17, Jane Newburger59, Kee Yuan Ngiam109, Wanjiku FM Njoroge110, James B Norman1, Jihad Obeid69, Marina P Okoshi102, Karen L Olson111, Gilbert S. Omenn20, Nina Orlova57, Brian D Ostasiewski112, Nathan P Palmer1, Nicolas Paris57, Lav P Patel8, Miguel Pedrera-Jiménez53, Emily R Pfaff113, Ashley C Pfaff114, Danielle Pillion1, Sara Pizzimenti36, Hans U Prokosch115, Robson A Prudente116, Andrea Prunotto21, Víctor Quirós-González53, Rachel B Ramoni117, Maryna Raskin42, Siegbert Rieg118, Gustavo Roig-Domínguez53, Pablo Rojo119, Paula Rubio-Mayo53, Paolo Sacchi58, Carlos Sáez120, Elisa Salamanca57, Malarkodi Jebathilagam Samayamuthu7, L. Nelson Sanchez-Pinto121, Arnaud Sandrin57, Nandhini Santhanam76, Janaina C.C Santos122, Fernando J Sanz Vidorreta24, Maria Savino123, Emily R Schriver124, Petra Schubert23, Juergen Schuettler125, Luigia Scudeller36, Neil J Sebire55, Pablo Serrano-Balazote53, Patricia Serre57, Arnaud Serret-Larmande126, Mohsin Shah55, Zahra Shakeri Hossein Abad1, Domenick Silvio127, Piotr Sliz66, Jiyeon Son128, Charles Sonday129, Andrew M South39, Anastasia Spiridou55, Zachary H. Strasser16, Amelia LM Tan1, Bryce W.Q. Tan5, Byorn W.L. Tan5, Suzana E Tanni102, Deanne M Taylor130, Ana I Terriza-Torres53, Valentina Tibollo28, Patric Tippmann81, Emma MS Toh34, Carlo Torti99, Enrico M Trecarichi99, Yi-Ju Tseng131, Andrew K Vallejos132, Gael Varoquaux133, Margaret E Vella1, Guillaume Verdy14, Jill-Jênn Vie134, Shyam Visweswaran7, Michele Vitacca135, Kavishwar B Wagholikar37, Lemuel R Waitman136, Xuan Wang1, Demian Wassermann63, Griffin M Weber1, Martin Wolkewitz81, Scott Wong5, Zongqi Xia4, Xin Xiong3, Ye Ye7, Nadir Yehya137, William Yuan1, Alberto Zambelli138, Harrison G Zhang1, Daniela Zöller21, Valentina Zuccaro58, Chiara Zucco101

1Department of Biomedical Informatics, Harvard Medical School, Boston, United States, 2Department of Electrical Computer and Biomedical Engineering, University of Pavia, Pavia, Italy, 3Department of Biostatistics, Harvard T.H. Chan School of Public Health, Boston, United States, 4Department of Neurology, University of Pittsburgh, Pittsburgh, United States, 5Department of Medicine, National University Hospital, Singapore, Singapore, Singapore, 6Department of Biostatistics and Bioinformatics, Duke University, Durham, United States, 7Department of Biomedical Informatics, University of Pittsburgh, Pittsburgh, United States, 8Department of Internal Medicine, Division of Medical Informatics, University Of Kansas Medical Center, Kansas City, United States, 9Department of Learning Health Sciences, University of Michigan Medical School, Ann Arbor, United States, 10Department of Biostatistics, Epidemiology, and Informatics, University of Pennsylvania Perelman School of Medicine, Philadelphia, United States, 11Institute for Biomedical Informatics, University of Pennsylvania Perelman School of Medicine, Philadelphia, United States, 12Department of Pediatrics, Harvard Medical School, Boston, United States, 13Michigan Institute for Clinical and Health Research (MICHR) Informatics, University of Michigan, Ann Arbor, United States, 14IAM unit, Bordeaux University Hospital, Bordeaux, France, 15Department of Neurology, Massachusetts General Hospital, Boston, United States, 16Department of Medicine, Massachusetts General Hospital, Boston, United States, 17Department of biomedical informatics, Hôpital Necker-Enfants Malade, Assistance Publique Hôpitaux de Paris (APHP), University of Paris, Paris, France, 18Department of Biomedical informatics, WiSDM, National University Health Systems Singapore, Singapore, Singapore, 19Department of Anaesthesia, National University Health Systems Singapore, Singapore, Singapore, 20Dept of Computational Medicine & Bioinformatics, Internal Medicine, Human Genetics, and School of Public Health, University of Michigan, Ann Arbor, United States, 21Institute of Medical Biometry and Statistics, Faculty of Medicine and Medical Center, University of Freiburg, Freiburg, Germany, 22Department of Ophthalmology, Mayo Clinic, Rochester, United States, 23Massachusetts Veterans Epidemiology Research and Information Center (MAVERIC), VA Boston Healthcare System, Boston, United States, 24Department of Medicine, David Geffen School of Medicine at UCLA, Los Angeles, United States, 25IT Department, Innovation & Data, APHP Greater Paris University Hospital, Paris, France, 26Division of Biomedical Informatics (Department of Internal Medicine), University of Kentucky, Lexington, United States, 27Department of Preventive Medicine, Northwestern University, Chicago, USA, 28Laboratory of Informatics and Systems Engineering for Clinical Research, Istituti Clinici Scientifici Maugeri SpA SB IRCCS, Pavia, Italy, 29Department of Electrical, Computer and Biomedical Engineering, University of Pavia, Pavia, Italy, 30Population Health and Data Science, MAVERIC, VA Boston Healthcare System, Boston, United States, 31IAM unit, INSERM Bordeaux Population Health ERIAS TEAM, Bordeaux University Hospital / ERIAS - Inserm U1219 BPH, Bordeaux, France, 32Unit of Internal Medicine and Endocrinology, Istituti Clinici Scientifici Maugeri SpA SB IRCCS, Pavia, Italy, 33Departments of Biomedical Informatics, Pediatrics, Cincinnati Children's Hospital Medical Center, University of Cincinnati, Cincinnati, United States, 34Yong Loo Lin School of Medicine, National University of Singapore, Singapore, Singapore, 35Department of Medicine, National University Health Systems Singapore, Singapore, Singapore, 36Scientific Direction, IRCCS Ca' Granda Ospedale Maggiore Policlinico di Milano, Milan, Italy, 37Department of Medicine, Massachusetts General Hospital, Boston, USA, 38BIOMERIS (BIOMedical Research Informatics Solutions), Pavia, Italy, 39Department of Pediatrics-Section of Nephrology, Brenner Children's, Wake Forest School of Medicine, Winston Salem, United States, 40Department of Biomedical Informatics, University of Kentucky, Lexington, United States, 41Department of Legal, Economic and Social Sciences, University Magna Graecia of Catanzaro, Catanzaro, Italy, 42Health Catalyst, INC., Cambridge, United States, 43Department of Surgery, ASST Pavia, Lombardia Region Health System, Pavia, Italy, 44Clinical Research Unit of Botucatu Medical School, São Paulo State University, Clinical Research Unit of Botucatu Medical School, São Paulo State University, Botucatu, Brazil, 45Pediatric emergency Department, Hôpital Necker-Enfants Malades, Assistance Public-Hôpitaux de Paris, Paris, France, 46National Center for Infectious Diseases, Tan Tock Seng Hospital, Singapore, Singapore, 47BIG-ARC, The University of Texas Health Science Center at Houston, School of Biomedical Informatics, Houston, United States, 48Department of Pediatrics, Medical University of South Carolina, Charleston, United States, 49Internal Medicine Department, Botucatu Medical School, São Paulo State University, Botucatu, Brazil, 50Department of Surgery, St. Luke's University Health Network, Bethlehem, United States, 51Department of Medicine, Division of Nephrology, Ente Ospedaliero Cantonale, Lugano, Switzerland, 52IT Department, ASST Pavia, Voghera, Italy, 53Health Informatics, Hospital Universitario 12 de Octubre, Madrid, Spain, 54Strategy and Transformation Department, APHP Greater Paris University Hospital, Paris, France, 55Digital Research, Informatics and Virtual Environments (DRIVE), Great Ormond Street Hospital for Children, UK, London, United Kingdom, 56North Carolina Translational and Clinical Sciences (NC TraCS) Institute, UNC Chapel Hill, Chapel Hill, United States, 57IT department, Innovation & Data, APHP Greater Paris University Hospital, Paris, France, 58Division of Infectious Diseases I, Fondazione I.R.C.C.S. Policlinico San Matteo, Pavia, Italy, 59Department of Cardiology, Boston Children's Hospital, Harvard Medical School, Boston, United States, 60Department of Biomedical Informatics, HEGP, APHP Greater Paris University Hospital, Paris, France, 61Department of Medical and Surgical Sciences, Data Analytics Research Center, University Magna Graecia of Catanzaro, Catanzaro, Italy, 62Department of Anesthesia, St. Luke's University Health Network, Bethlehem, United States, 63Université Paris-Saclay, Inria, CEA, Palaiseau, France, 64INRIA Sophia-Antipolis – ZENITH team, LIRMM, Montpellier, France, 65Department of Internal Medicine, University of Kentucky, Lexington, United States, 66Computational Health Informatics Program, Boston Children's Hospital, Boston, United States, 67UOC Ricerca, Innovazione e Brand reputation, ASST Papa Giovanni XXIII, Bergamo, Bergamo, Italy, 68Informatics Institute, University of Alabama at Birmingham, Birmingham, United States, 69Biomedical Informatics Center, Medical University of South Carolina, Charleston, United States, 70Clinical Research Informatics, Boston Children's Hospital, Boston, United States, 71IT department, Innovation & Data (APHP), UMRS1142 (INSERM), APHP Greater Paris University Hospital, INSERM, Paris, France, 72Department of Biomedical and Health Informatics, Children's Hospital of Philadelphia, Philadelphia, United States, 73VA Informatics and Computing Infrastructure, VA Salt Lake City Health Care System, Salt Lake City, United States, 74SED/SIERRA, Inria Centre de Paris, Paris, France, 75Health Information Technology & Services, University of Michigan, Ann Arbor, United States, 76Heinrich-Lanz- Center for Digital Health, University Medicine Mannheim, Heidelberg University, Mannheim, Germany, 77Department of Computational Biology and Bioinformatics, University of Michigan, Ann Arbor, United States, 78Biostatistics, Perelman School of Medicine at the University of Pennsylvania, Philadelphia, United States, 79Department of Anesthesiology, Critical Care, and Pain Medicine and Computational Health Informatics Program, Boston Children's Hospital, Boston, United States, 80Institute of Digitalization in Medicine, Faculty of Medicine and Medical Center, University of Freiburg, Freiburg, Germany, 81Institute of Medical Biometry and Statistics, Institute of Medical Biometry and Statistics, Medical Center, University of Freiburg, Freiburg, Germany, 82Department of Preventive Medicine, Northwestern University, Chicago, United States, 83Department of Biomedical Informatics, HEGP, APHP Greater Paris University Hospital, Paris, France, 84Center for Precision Psychiatry, Massachusetts General Hospital, Boston, United States, 85Medical University of South Carolina, Charleston, United States, 86Department of Pediatrics, Division of Human Genetics, The Children's Hospital of Philadelphia and the Perelman School of Medicine at the University of Pennsylvania, Philadelphia, United States, 87Center for Medical Information and Communication Technology, University Hospital Erlangen, Germany, 88Renaissance Computing Institute/Department of Computer Science, University of North Carolina, Chapel Hill, Chapel Hill, United States, 89Clinical Research Unit, Saint Antoine Hospital, APHP Greater Paris University Hospital, Paris, France, 90Clevy.io, Paris, France, 91Department of Biostatistics, Harvard T. H. Chan School of Public Health, Boston, United States, 92Department of Anaesthesia, National University Health Systems, Singapore, Singapore, Singapore, 93Department of Biostatistics, Epidemiology and Informatics, University of Pennsylvania Perelman School of Medicine, Philadelphia, United States, 94Harvard Catalyst, Harvard Medical School, Boston, United States, 95Clinical Research Unit, Paris Saclay, APHP Greater Paris University Hospital, Boulogne-Billancourt, France, 96Medical Informatics Center, Hospital das Clínicas, Faculty of Medicine of Botucatu, Clinical Research Unit of Botucatu Medical School, São Paulo State University, Botucatu, Brazil, 97Department of Surgery, Beth Israel Deaconess Medical Center, Boston, United States, 98Department of Anesthesiology and Critical Care, Children's Hospital of Philadelphia, Philadelphia, United States, 99Department of Medical and Surgical Sciences, Infectious and Tropical Disease Unit, University Magna Graecia of Catanzaro, Catanzaro, Italy, 100ENS, PSL University, Paris, France, 101Department of Medical and Surgical Sciences, University Magna Graecia of Catanzaro, Catanzaro, Italy, 102Internal Medicine Department of Botucatu Medical School, São Paulo State University, Botucatu, Brazil, 103Department of Biomedical Health Informatics, Children's Hospital of Philadelphia, Philadelphia, United States, 104Department of Computational Biomedicine, Cedars-Sinai Medical Center, West Hollywood, United States, 105Pediatric Infectious Disease Department, Hospital Universitario 12 de Octubre, Madrid, Spain, 106Department of Biostatistics, Epidemiology, and Informatics, Institute for Biomedical Informatics,, University of Pennsylvania Perelman School of Medicine, Berwyn, United States, 107Department of Infectious Diseases, Great Ormond Street Hospital for Children, UK, London, United Kingdom, 108Harvard Catalyst | The Harvard Clinical and Translational Science Center, Harvard Medical School, Boston, United States, 109Department of Biomedical informatics, WiSDM, National University Health System Singapore, Singapore, Singapore, 110Department of Psychiatry, University of Pennsylvania Perelman School of Medicine, Philadelphia, United States, 111Computational Health Informatics Program and Department of Pediatrics, Boston Children's Hospital, Harvard Medical School, Boston, United States, 112CTSI, WFBMI, Wake Forest School of Medicine, Winston Salem, United States, 113NC TraCS Institute, UNC Chapel Hill, Chapel Hill, United States, 114Department of Surgery, Beth Israel Deaconess Medical Center, Harvard Medical School, Boston, United States, 115Department of Medical Informatics, University of Erlangen-Nürnberg, Erlangen, Germany, 116Clinical Research Unit São Paulo State University, Brazil, Clinical Research Unit of Botucatu Medical School, São Paulo State University, Botucatu, Brazil, 117Office of Research and Development, Department of Veterans Affairs, Washington, DC, United States, 118Division of Infectious Diseases, Department of Medicine II, Medical Center – University of Freiburg, Faculty of Medicine, Freiburg, Germany, 119Pediatric Infectious Disease Deparment, Hospital Universitario 12 de Octubre, Madrid, Spain, 120Biomedical Data Science Lab, ITACA Institute, Universitat Politècnica de València, Spain, Valencia, Spain, 121Department of Pediatrics (Critical Care), Northwestern University Feinberg School of Medicine, Chicago, United States, 122Nurse departament of FMB - medicine school of Botucatu, Clinical Research Unit of Botucatu Medical School, São Paulo State University, Botucatu, Brazil, 123ASST Pavia, Lombardia Region Health System, Management Engineer, Direction, Pavia, Italy, 124Data Analytics Center, University of Pennsylvania Health System, Philadelphia, United States, 125Department of Anesthesiology, University Hospital Erlangen, FAU Erlangen-Nürnberg, Erlangen, Germany, 126Hôpital Saint Louis, Department of Biostatistics and Bioinformatics, APHP Greater Paris University Hospital, Paris, France, 127MICHR Informatics, University of Michigan, Ann Arbor, United States, 128Department of Neurology, University of Pittsburgh Medical Center, Pittsburgh, United States, 129Critical Care Medicine, Department of Medicine, St. Luke's University Health Network, Bethlehem, United States, 130Department of Biomedical Health Informatics and the Department of Pediatrics, The Children's Hospital of Philadelphia and the University of Pennsylvania Perelman Medical School, Philadelphia, United States, 131Department of Information Management, National Central University, Taoyuan, Taiwan, 132Clinical & Translational Science Institute, Medical College of Wisconsin, Milwaukee, United States, 133Université Paris-Saclay, Inria, CEA, Montréal Neurological Institute, McGill University, Palaiseau, France, 134SequeL, Inria Lille, Villeneuve-d'Ascq, France, 135Respiratory Department, ICS S. Maugeri IRCCS Pavia Italy, Lumezzane (BS), ITALY, 136Department of Health Management and Informatics, University of Missouri, Columbia, Columbia, United States, 137Department of Anesthesiology and Critical Care Medicine, Children's Hospital of Philadelphia and University of Pennsylvania, Philadelphia, United States, 138Department of Oncology, ASST Papa Giovanni XXIII, Bergamo, Bergamo, Italy.
